# Parasitological Clearance Rates and Drug Concentrations of a Fixed Dose Combination of Azithromycin-Chloroquine in Asymptomatic Pregnant Women with Plasmodium Falciparum Parasitemia: An Open-Label, Non-Comparative Study in Sub-Saharan Africa

**DOI:** 10.1371/journal.pone.0165692

**Published:** 2016-11-18

**Authors:** Kamija Phiri, Joshua Kimani, George A. Mtove, Qinying Zhao, Ricardo Rojo, Jeffery Robbins, Stephan Duparc, Ayman Ayoub, Pol Vandenbroucke

**Affiliations:** 1 College of Medicine, University of Malawi, Blantyre, Malawi; 2 College of Health Sciences, University of Nairobi, Nairobi, Kenya; 3 National Institute for Medical Research, Amani Medical Research Centre, Tanga, Tanzania; 4 Pfizer Inc., Groton, Connecticut, United States of America; 5 Medicines for Malaria Venture, Geneva, Switzerland; 6 Pfizer Inc., Sandwich, Kent, United Kingdom; 7 Pfizer Inc., New York, New York, United States of America; University of California Los Angeles, UNITED STATES

## Abstract

**Background:**

Malaria remains one of the most important causes of morbidity and mortality in pregnant women and their newborn babies in sub-Saharan Africa. Intermittent preventive treatment in pregnancy (IPTp) is recommended by the World Health Organization (WHO) to reduce the burden of disease and improve maternal and neonatal survival and general health. Due to the growing resistance to sulfadoxine-pyrimethamine (SP), the current WHO-recommended drug for IPTp, identification of new and effective drugs is an urgent priority.

**Methods and Findings:**

This was an open-label, non-comparative study (NCT01103713) in 5 countries in East and sub-Saharan Africa (Benin, Kenya, Malawi, Tanzania, and Uganda) to assess parasitological response and drug concentrations of a single, 3-day course of four tablets of a fixed-dose combination of azithromycin-chloroquine (AZCQ) 250/155 mg given during the second or third trimester to women with asymptomatic *Plasmodium falciparum* parasitemia in their first or second pregnancy. Parasitemia was determined by microscopy and molecular genotyping was performed to characterize parasites relative to the baseline infection. Weekly follow-up visits took place until day 42 after first dose and additional follow-up occurred after delivery. Systemic concentrations of azithromycin (AZ), chloroquine (CQ), and the CQ metabolite, desethyl CQ (DECQ) were evaluated at Day 0 (pre-dose), at Day 2 (pre-dose, 2 and 8 hours) and randomly at Days 7 and 14. Systemic concentrations of CQ and DECQ were also measured randomly at Day 21 and Day 28. In total, 404 women were screened for eligibility and 168 were treated, 155 of whom completed the study. PCR-adjusted parasitological response in the modified intent-to-treat population at day 28 (the primary efficacy endpoint) was estimated by the Kaplan-Meier method as 99.35% (95% confidence interval [CI]: 97.76, 100.00). PCR-adjusted parasitological response remained high at day 42 (95.19%; 95% CI: 91.35, 99.03). In general, the mean concentrations of serum AZ, plasma CQ, and plasma DECQ showed large CV% values (ranges of 33–156%, 42–228%, and 57–109%, respectively). There were 157 live births, three stillbirths, and eight pregnancies of unknown outcome: 7 due to withdrawal of participant consent and 1 lost to follow-up. The most frequent treatment-emergent adverse events were vomiting (20.8%) and dizziness (19.6%).

**Conclusions:**

These results suggest that a 3-day course of AZCQ can lead to an adequate 28-day parasitological response.

## Introduction

Malaria is a serious infection caused by protozoan parasites of the *Plasmodium* species that are transmitted when infected female anopheline mosquitoes bite a human host to consume a blood meal [1]. The characteristic clinical signs and symptoms of malaria include recurrent attacks of fever, paroxysm, hemolytic anemia, and jaundice [2]. Asymptomatic malaria, in which individuals are infected but exhibit few signs and symptoms of disease presents substantial challenges in terms of eradication or control strategies [3].

Although malaria cases occur in all the major tropical and sub-tropical regions of the world, Africa has the greatest number of people at risk of contracting the disease and has the greatest number of deaths related to malaria [4]. The World Health Organization (WHO) World Malaria Report 2013 states that there were an estimated 207 million cases of malaria worldwide in 2012, with 80% of these estimates attributed to Africa [4]. There were 627,000 estimated deaths due to malaria, 90% of which were estimated to occur in Africa. Approximately 482,000 of these deaths were estimated to occur in children below 5 years of age, representing 77% of all global malaria deaths [4]. Within Africa, the majority of malaria cases occur in sub-Saharan countries, and while some malaria infection control measures are in place across most of the region, transmission and mortality rates remain high and this results in an enormous burden on health care systems [4].

Pregnant women are more susceptible to malaria, and susceptibility is greatest in the first and second pregnancy [5–7]. Malaria in pregnancy is, therefore, one of the most common causes of preventable mortality and morbidity in pregnant women and infants in sub-Saharan Africa [8]. There are an estimated 30 million pregnancies that are at risk of malaria infection across the region each year; and approximately 200,000 infants and 10,000 women die of complications related to malaria in pregnancy annually [9,10].

Intermittent preventive treatment in pregnancy (IPTp) is an important means of reducing the detrimental health impact of malaria on pregnant mothers and their unborn children [11]. The WHO recommends IPTp with sulfadoxine-pyrimethamine (SP) at each ante-natal visit after the first trimester in moderate and high-malaria transmission regions [11]. Nevertheless, resistance to SP is widespread, especially in East Africa, and evidence from a study in Burkina Faso has shown that the use of SP in IPTp led to an increase in the prevalence of resistant infections from 1.3% in 2000 to 35.3% in 2009, and 54.3% in 2011 (*p*≤0.001) [12]. An additional concern is that SP is unsafe for administration during the first trimester of pregnancy [13–15]. There is, therefore, a need for new drugs for IPTp.

The combination of azithromycin (AZ) and chloroquine (CQ) was evaluated as a potential alternative to SP for IPTp in another study (NCT01103063) [9]. AZ and CQ have been used extensively in pregnant women during all trimesters. AZ has been used for the treatment and prevention of sexually transmitted infections including *Neisseria gonorrhoeae* and *Chlamydia trachomatis* infections [16]. The combination of AZ and CQ (AZCQ) has been shown to produce synergistic reductions in malaria parasite burden *in vitro* and in animal model studies [17,18].

Co-administration of AZ and CQ has demonstrated efficacy, safety, and tolerability in the treatment of symptomatic uncomplicated malaria in two multi-country Phase 3 clinical studies in non-pregnant adults (NCT00082576 and NCT00367653) [19] and one study in children (NCT00677833) [20].

The study we describe here (NCT01103713) was designed to assess weekly parasitological response and drug concentrations of a single fixed-dose combination of AZCQ administered over 3 days in women with asymptomatic *Plasmodium falciparum* parasitaemia, in their first or second pregnancy, in sub-Saharan Africa. The aim of this study was to determine if AZCQ could clear the malaria parasites from the blood during pregnancy with a similar efficacy as that demonstrated in previous adult treatment studies. However, unlike typical treatment studies that rely on demonstrating adequate clinical and parasitological response as a primary endpoint [21], the trial was designed to provide supportive data in asymptomatic, parasitemic women for a Phase 3 IPTp companion study (NCT01103063). An out-patient design was used to reflect normal antenatal care conditions for IPTp programs.

## Methods

### Study Design and Patients

This was an open-label, non-comparative out-patient study in primi- or secundigravidae women ≥16 to ≤35 years of age in the second or third trimesters of pregnancy with asymptomatic peripheral *P*. *falciparum* parasitemia (counts of 80–100,000 cells/μl). Study participants received a single 3-day course of AZCQ IPTp therapy after providing informed consent.

Women were excluded if they were revealed to have multiple gestations as per ultrasound screening, had any chronic illness that might have adversely affected fetal growth or viability, or had evidence of current obstetric complications that could adversely impact the pregnancy or fetal outcomes, including presence of congenital anomalies, placenta previa, or abruption. Additionally, women were excluded if they had clinical signs and symptoms of malaria, history of fever within prior 24 h, baseline hemoglobin <8 g/dl, or had used antimalarial drugs in the previous 4 weeks. An inability to tolerate oral treatment in tablet form, or a known allergy to the study drugs (AZ, CQ, and SP, as these treatments are continued after study completion) or to any macrolides or sulfonamides, use of any medication that may have interfered with the evaluation of the study drug or that was contra-indicated during pregnancy, a current history of smoking or alcohol/drug abuse, or severe acute or chronic medical or psychiatric condition or laboratory abnormality (e.g., clinically-assessed symptomatic HIV infection, sickle-cell disease, renal, or hepatic disease) were also reasons for exclusion.

A fixed-dose AZCQ combination (four fixed dose combination tablets of AZ 250 mg/CQ 155 mg) was used in this study [22]. Two tablets of this formulation had previously been shown to result in comparable systemic exposures of AZ and CQ to those achieved by administration of standalone tablets of AZ (Zithromax^®^ 500 mg) and CQ (Aralen^®^ 300 mg) [20]). The first dose on Day 0 and the third dose on Day 2 were administered under supervision during the antenatal care visit; while the second dose on Day 1 was administered at home by a field worker. After administration of study drug, weekly follow-up visits took place until day 42 and additional follow-up took place at or after delivery. Long-lasting insecticide-treated bed nets were provided on day 0 and their installation was verified during a day 1 home visit by field workers.

The study was conducted in accordance with the Declaration of Helsinki on Ethical Principles for Medical Research Involving Human Subjects, adopted by the General Assembly of the World Medical Association (1996), and was conducted in accordance with the International Conference on Harmonization (ICH) guideline on Good Clinical Practice (GCP), and applicable local regulatory requirements and laws. The study protocol was approved by the Independent Ethics Committee (IEC) of London School of Hygiene and Tropical Medicine and the following local Institutional Review Board (IRB)/IECs: the Comité National d’Ethique pour la recherché en Sante in Cotonou, Benin; the Kenyatta National Hospital—University of Nairobi Ethics Review Committee in Nairobi, Kenya; the College of Medicine Research and Ethics Committee in Blantyre, Malawi; the Medical Research Coordinating Committee in Dar es Salaam, Tanzania; and the Uganda National Council of Science and Technology, the School of Medicine Research and Ethics Committee of Makerere University, and the Mulago Hospital Research and Ethics Committee in Kampala, Uganda. An independent External Data Monitoring Committee (EDMC) oversaw the conduct of the study. Written informed consent (or assent from participants <18 years of age) was obtained from all study participants or their legally-acceptable representatives prior to any study-related procedure.

### Primary and Secondary Endpoints

Parasitological response was assessed by analysis of peripheral blood smears (thick and thin) stained by standard Giemsa staining for parasite identification. Parasite numbers were counted using the white blood cell counting method on thick smears. Each blood smear was read by two microscopists blinded to the reading of each other. In the event of any discrepancies, a third microscopist read the blood smear and the average of the two most concordant results was used. A positive blood smear had a *P*. *falciparum* count 80–100,000 μl upon examination of 100 high power fields on thick smear. A blood slide was considered negative when the examination of 100 high power fields on the thick smear do not show the presence of any *P*. *falciparum* parasites.

Molecular testing by polymerase chain reaction (PCR) was used to verify the genotype of asexual *P*. *falciparum* parasites [23]. Analysis of PCR-adjusted data allowed the differentiation between the reappearance of the same genotypic parasite as present at baseline (considered a treatment failure, recrudescence) and re-infection with genotypically different *P*. *falciparum* after parasitological cure of the baseline infection. Conversely, analysis of PCR-unadjusted data made no distinction between reappearance of the baseline infection or re-infection with a genotypically distinct parasite, both of which would be considered treatment failures. The primary endpoint was the PCR-adjusted parasitological cure rate on day 28 following a single 3-day dosing regimen of AZCQ in asymptomatic pregnant women with *P*. *falciparum* parasitemia. Secondary endpoints included PCR-adjusted and unadjusted parasitological cure rates on days 7, 14, 21, 28 (unadjusted), 35, and 42 post first dose of study drug, and parasite counts (number of asexual *P*. *falciparum* parasites per μl of blood) at each visit.

### Safety Endpoints

Adverse events (AEs), reported in mothers and neonates, were analyzed. Additionally, temperature, physical examinations, hemoglobin concentrations, and exposure *in utero* were recorded. An AE with onset after the first dose of study drug was to be counted as a treatment emergent adverse event (TEAE) if it occurred on or before 35 days post last dose. If the onset was prior to the first dose of study drug and the severity increased thereafter, the event was also to be counted as a TEAE.

### Statistical Analysis

Study populations were defined as follows. The intent to treat (ITT) analysis population consisted of all participants who received at least one dose of study drug and who had baseline blood smears positive for *P*. *falciparum* monoinfection. The modified ITT (MITT) comprised a subset of the ITT population who had *P*. *falciparum* monoinfection (confirmed by microscopy) with parasite counts in the range of 80–100,000/μl on thick blood smears. The per protocol (PP) analysis population comprised a subset of MITT participants who received all 3 days of study drug. Safety analysis data included all mothers who received at least one dose of study drug, while for neonates it consisted of all liveborn babies. Parasite counts were summarized descriptively using the sample mean, standard deviation (SD), standard error (SE), median, minimum, and maximum. Primary and secondary parasitological endpoints were estimated from the Kaplan-Meier (KM) curve (primary analysis), with corresponding 95% confidence interval (CI), based on time to the first occurrence of parasitological failure. If, at a given time point, no parasitological failures had yet occurred, resulting in a missing variance estimate, an exact CI derived from the binomial distribution was computed. A subject was a parasitological responder if she had a zero parasite count on the day 7 visit without subsequent recurrence through the day of consideration (PCR adjusted or unadjusted as applicable), otherwise she was a parasitological failure. Participants who did not experience the defined event (parasitological failure) were considered censored. Crude incidence of parasite cure was also calculated for days 28 and 42. A sensitivity analysis was also performed considering study discontinuations as failures. Data were pooled across all sites for analysis. No statistical hypothesis was tested.

### Drug Concentrations

Concentrations of serum AZ, plasma CQ, and the CQ metabolite, desethyl-CQ (DECQ) were evaluated with sparse pharmacokinetic sampling on day 0 predose, day 2 predose, 2 h (as close to 2 h as possible), and 8 h (time window: 4–12 h) post dose, and at a random time point on days 7 and 14. In addition, due to the long half-life of CQ, plasma concentrations of CQ and DECQ were measured at a random time point on days 21 and 28.

Blood samples were centrifuged at 1700 rpm for 10 minutes at 4°C. Serum for AZ or plasma for CQ and DECQ samples were stored within 1 hour of collection at -20°C until shipped for assay. AZ, CQ, and DECQ concentrations were analyzed using validated, sensitive, and specific high-performance liquid chromatography tandem mass spectrometric methods in compliance with Pfizer standard operating procedures. AZ assays were performed by Bioanalytical Laboratory (BASi) (USA) and CQ/DECQ assays were performed by Bioanalytical Laboratory WuXi appTec (China).

## Results

### Patients Characteristics and Populations Analyzed

**Fig 1:**
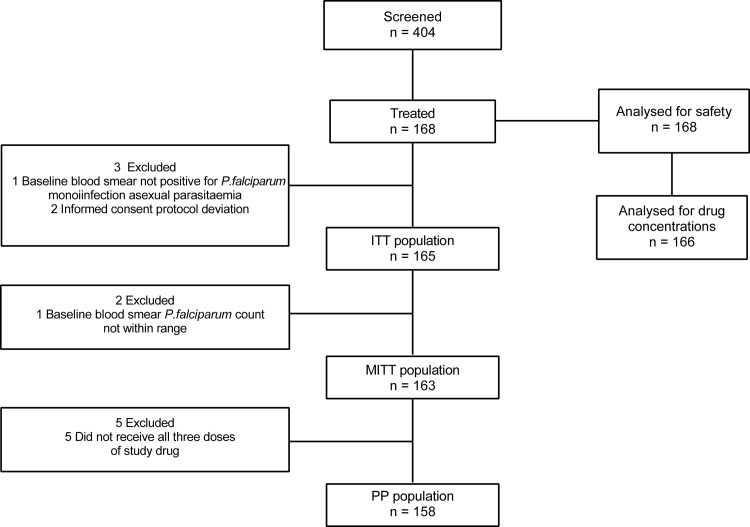
Study Design. ITT, intent-to-treat; MITT, modified ITT; PP, per protocol.

The study was conducted between March 2011 and October 2013 in six study sites in five countries: Benin, Kenya, Malawi, Tanzania, and Uganda. In total, 404 women were screened, of whom, 168 were treated and 155 completed the study (Fig 1). The site by site breakdown of study completion is as follows: in Kenya, 62/67 (92.5%) participants completed the study; in Tanzania (2 sites), 1/1 (100.0%) participant; in Benin, 1/1 (100.0%) participant; in Uganda, 46/49 (93.9%) participants; and in Malawi, 45/50 (90.0%) participants.

Reasons for discontinuation included unwillingness to continue study participation (eight women), lost to follow-up (two women), termination of the study (two women), and another due to relocation (one woman). The safety analysis set comprised all 168 women who were treated and 157 live-born neonates. There were three stillbirths recorded, and eight pregnancy outcomes were not recorded because 7 participants withdrew consent and 1 was lost to follow-up. Drug concentrations were assessed from samples from 166 participants. The MITT population comprised 163 women who had a *P*. *falciparum* monoinfection (confirmed by microscopy) with parasite counts between 80 and 100,000 per μl (one subject in Tanzania who had a parasite count of 500,000 per μl was incorrectly included in the study) and who received at least one dose of study drug. In addition, two participants were found to have been improperly consented and were, therefore, excluded from the efficacy analysis. The PP analysis set comprised 158 participants in the MITT population who received all three daily doses of study drug.

Baseline demographics for the safety population are shown in Table 1. For 124 (73.8%) women this was the first pregnancy, while 43 (25.6%) women had one previous pregnancy resulting in a live birth, and one woman had a previous pregnancy that resulted in a spontaneous abortion (Table 1). One woman had two prior pregnancies (a protocol deviation), both resulting in a live birth. Most women with one or more prior pregnancies reported vaginal deliveries (40 [23.8%] women in the group with one prior pregnancy and one [0.6%] woman in the group with two prior pregnancies). A cesarean section was reported for two (1.2%) women in the group with one prior pregnancy. No participant reported previous stillbirth, elective terminations, ectopic or molar pregnancies, history of subfertility, risk factors for adverse pregnancy outcomes due to environmental/occupational exposures, or a family history of congenital abnormality/genetic diseases. Long-lasting insecticide-treated bed nets were installed for 164/165 participants in the ITT population. The median baseline asexual parasite count for all participants in the ITT population was 1240 per μl (Table 2).

**Table 1:** Baseline demographics and summary of obstetric medical history in the safety population.

Characteristics	AZCQ (N = 168)
**Age, years**	
Mean (SD)	18.8 (2.3)
Range	16–34
**Weight, kg**	
Mean (SD)	56.6 (7.1)
Range	36.0–79.5
**Body mass index, kg/m**^**2**^	
Mean (SD)	22.5 (2.3)
Range	15.6–29.9
**Height, cm**	
Mean (SD)	158.6 (6.5)
**Number of prior pregnancies**	
0	124 (73.8)
1	43 (25.6)
2	1 (0.6)
Total	168
**Number of prior live births**	
0	125 (74.4)
1	42 (25.0)
2	1 (0.6)
Total	168
**Number of prior spontaneous abortions**	
0	167 (99.4)
1	1 (0.6)
2	0
Total	168
**Number of prior vaginal deliveries**	
0	127 (75.6)
1	40 (23.8)
2	1 (0.6)
Total	168
**Number of prior Cesarean sections**	
0	166 (98.8)
1	2 (1.2)
2	0
Total	168
**Number of prior obstetric complications**	
0	167 (99.4)
1	1 (0.6)
2	0
Total	168
**Other obstetric history**	
Yes	2 (1.2)
No	166 (98.8)
Total	168
**Age of subject at first delivery (years)**	
Not applicable	124 (73.8)
<19	28 (16.7)
19–25	15 (8.9)
26–30	1 (0.6)
>31	0
Total	168

AZCQ, azithromycin-chloroquine; SD, standard deviation.

**Table 2:** Baseline asexual parasite count.

	AZCQ (N = 165)
Mean	6693
SD	41951
SE	3266
CV (%)	627
Median	1240
Minimum	48
Maximum	535000

AZCQ, azithromycin-chloroquine; CV, coefficient of variation; SD, standard deviation. Parasite counts are represented as per μl.

### CQ Resistance

In the ITT population, 100 (60.6%) participants had infections with parasites carrying a mutant version of either one or both of the *P*. *falciparum* CQ resistance transporter (*PfCRT*) or *P*. *falciparum* multidrug resistance 1 (*Pfmdr1*) genes. The remaining 65 (39.4%) participants had wild-type infections.

### Efficacy

At day 28, 153 of 154 participants in the PP population had a PCR-adjusted parasitological response (KM estimate: 99.35%; 95% CI: 97.76, 100.00) (Tables 3 and 4). Results for the MITT population (data not shown) were identical. Sensitivity analyses for PCR-adjusted parasitological response (in which study dropouts were considered failures) supported these results, with 153/160 participants (95.69% [CI: 92.26, 99.13]) assessed as parasitological responders on day 28 in the MITT population and 153/155 participants (98.72% [CI: 96.64, 100.00]) in the PP population. Results for the ITT analysis population (data not shown) were similar to those for the PP and MITT populations.

**Table 3:** Crude estimates of parasitological responders at days 28 and 42 in the PP analysis population.

	PP population (N = 158)	
	Day 28	Day 42
**PCR-adjusted**		
Recrudescence	1/154 (0.65%)	7/138 (5.07%)
No recrudescence (cure)	153/154 (99.35%)	131/138 (94.93%)
Reinfection*	6/154	13/138
Prior study discontinuation^†^	1	1
Missing blood smears^†^	3	4
Reinfection prior to visit*^,^^†^	0	15
**PCR-unadjusted**		
Recurrent parasitemia	7/154 (4.55%)	33/152 (21.71%)
No recurrent parasitemia (cure)	147/154 (95.45%)	119/152 (78.29%)
Received antimalarial at the visit*	0/154	1/152
Prior study discontinuation^†^	1	1
Missing blood smears^†^	3	4
Received antimalarial prior to visit*^,^^†^	0	1

*One subject at visit day 42 and 1 subject prior to the visit had blood smears done, treated with an antimalarial based on symptoms, but later found to have no parasitemia.

^†^Excluded from analysis.

PCR, polymerase chain reaction; PP, per-protocol.

**Table 4:** Kaplan-Meier estimates of the percentage parasitological responders at days 7–42 in the PP analysis population.

	PP population (N = 158)	
	PCR-adjusted	PCR-unadjusted
	n, estimate, (95% CI)	n, estimate, (95% CI)
Day 7	156, 100.00	156, 100.00
	(97.66, 100.00)	(97.66, 100.00)
Day 14	154, 100.00	154, 100.00
	(97.63, 100.00)	(97.63, 100.00)
Day 21	154, 100.00	154, 100.00
	(97.63, 100.00)	(97.63, 100.00)
Day 28	154, 99.35	154, 95.45
	(97.76, 100.00)	(91.84, 99.07)
Day 35	148, 96.65	154, 87.66
	(93.42, 99.87)	(82.14, 93.18)
Day 42	138, 95.19	152, 78.43
	(91.35, 99.03)	(71.59, 85.28)

n = N minus the number censored per time point._

CI, confidence interval for the estimate; PCR, polymerase chain reaction; PP, per protocol.

Day 28 results for PCR-adjusted parasitological response in the PP population by site were: Kenya 100%, Benin 100%, Uganda 97.73%, Malawi 100%. Day 42 results for PCR-adjusted parasitological response in the PP population by site were: Kenya 96.35%, Benin 100%, Uganda 87.91%, and Malawi 100%.

Results for PCR-unadjusted data were identical to PCR-adjusted data until day 21, and were slightly lower, but still above 95%, at day 28. By days 35 and 42, PCR-unadjusted results were markedly lower, reaching approximately 78% at the end of the follow-up period (Table 4).

### Safety

There were no deaths reported in the pregnant mothers participating in this study. There were four deaths in neonates due to AEs: two neonatal asphyxia, one sudden infant death syndrome, and one premature baby (no further details recorded). None of these were considered related to study drug.

No mothers experienced serious AEs (SAEs) and nine SAEs were reported in neonates. TEAEs were reported in 92 (54.8%) mothers and 27 (17.2%) neonates. The most common TEAEs, those occurring in at least 5 mothers, are listed in Table 5. The most frequent TEAEs were vomiting (20.8%) and dizziness (19.6%). No mothers and seven (4.5%) neonates experienced severe AEs. Treatment-related TEAEs were reported in 71 (42.3%) mothers, and none in neonates.

**Table 5:** All-causality treatment-emergent adverse events occurring in ≥5 mothers.

Adverse Event*	AZCQ (N = 168)
Vomiting	35 (20.8)
Dizziness	33 (19.6)
Pruritus	13 (7.7)
Infection parasitic**	12 (7.1)
Headache	10 (6.0)
Generalized pruritus	9 (5.4)
Malaria	8 (4.8)
Fatigue	7 (4.2)
Upper respiratory infection	7 (4.2)
Nausea	6 (3.6)

Participants were counted only once per treatment in each row.

AZCQ, azithromycin-chloroquine.

*System organ class preferred term (MedDRA)

**an infection adverse event that results in infection by a parasite.

### Drug Concentrations

Mean serum AZ concentrations were 194, 994, and 708 ng/ml at 0, 2, and 8 h on day 2, respectively, and 54.4 and 20.3 ng/ml on days 7 and 14, respectively (Table 6). Mean plasma CQ concentrations were: 306, 621, and 641 ng/ml at 0, 2, and 8 h on day 2, respectively; and 130, 43.1, 22.4, and 12.7 ng/ml on days 7, 14, 21, and 28, respectively. Mean plasma DECQ concentrations were: 184, 220, and 242 ng/ml at 0, 2, and 8 h on day 2, respectively; and 144, 55.5, 29.8, and 19.4 ng/ml on days 7, 14, 21, and 28, respectively (Table 6). In general, the mean concentrations of serum AZ, plasma CQ, and plasma DECQ showed large CV% values (ranges of 33–156%, 42–228%, and 57–109%, respectively).

**Table 6:** Summary of drug concentrations (ng/ml).

Study Day	Planned Time Post Dose* (h)	Serum AZ^†^Mean (CV%)	Plasma CQ^†^Mean (CV%)	Plasma DECQ^†^Mean (CV%)
0	0	0	0	0
		0n = 161	0n = 160	0n = 158
2	0	194 (32.9)	306 (42.2)	184 (64.7)
		n = 158	n = 158	n = 158
2	2	994 (55.5)	621 (53.1)	220 (59.2)
		n = 147	n = 147	n = 147
2	8	708 (46.2)	641 (46.5)	242 (57.0)
		n = 159	n = 159	n = 159
7	HNS	54.4 (44.7)	130 (71.0)	144 (85.8)
		n = 155	n = 155	n = 155
14	HNS	20.3 (156)	43.1(104)	55.5 (98.7)
		n = 153	n = 154	n = 154
21	HNS	-	22.4 (209)	29.8 (109)
			n = 156	n = 156
28	HNS	-	12.7 (228)	19.4 (100)
			n = 156	n = 156

*Planned time post dose = planned time post last dose before the PK sample collection.

^†^Arithmetic mean.

AZ, azithromycin; CQ, chloroquine; CV, coefficient of variation; DECQ, desethylchloroquine; HNS, hour not specified.

### Birth Outcomes

Of the 157 neonates, 66 (42.0%) were female and 91 (58.0%) were male. The mean birth length (SD) was 46.3 cm (3.6) and the mean birth weight (SD) was 3022.2 g (494.5), while nine of the 137 with recorded birth weight (6.6%) had low birth weight (LBW; <2500 g). There were 144 (91.7%) normal newborns. Congenital malformations or anomalies were reported for two (1.3%) newborns (one hypospadias and one polydactyly), and other neonatal problems or abnormalities were reported for nine (5.7%) newborns. The outcome for two (1.3%) newborns was unknown (Tables 7 and 8).

**Table 7:** Summary of pregnancy outcomes in the safety population.

Pregnancy Outcomes	AZCQ (N = 168)
**Location of delivery**	
Medical facility	130 (81.3)
Home	27 (16.9)
Other	3 (1.9)
Total	160
**Mode of delivery**	
Vaginal	145 (90.6)
Cesarean section	15 (9.4)
Total	160
**Delivery assisted by trained obstetric personnel**	
Yes	132 (82.5)
No	27 (16.9)
Total	160*
**Labor induced?**	
Yes	3 (1.9)
No	155 (96.9)
Total	160*
Complications during delivery?**	
Yes	42 (26.3)
No	117 (73.1)
Total	160*
**Outcome of birth**	
Full term live birth	151 (94.4)
Premature birth	6 (3.8)
Spontaneous abortion	0
Induced/elective abortion	0
Stillbirth	3 (1.9)
Unknown	0
Total	160

AZCQ, azithromycin-chloroquine.

*Outcome was unknown for one or more subjects.

**Pre-eclampsia, hemorrhage, or infection.

**Table 8:** Summary of neonate outcomes at birth in the safety population.

	AZCQ (N = 157*)
**Number (%) of neonates**	
**Gender**	
Female	66 (42.0)
Male	91 (58.0)
**Birth length (cm)**	
Mean (SD)	46.3 (3.6)
Range	29.0–54.0
N	133 (84.7)
**Birth weight (g)**	
Mean (SD)	3022.3 (494.5)
Range	1200.0–4900.0
N	137 (87.3)
**Head circumference (cm)**	
Mean (SD)	34.3 (2.2)
Range	24.0–46.0
N	133 (84.7)
**APGAR score (5 min)**	
Mean (SD)	9.7 (1.2)
Range	1.0–10.0
N	126 (80.3)
Normal newborn**	
Yes	144 (91.7)
No	11 (7.0)
Total	155
**Congenital malformation/anomaly**	
Yes (1 hypospadia, 1 polydactyly)	2 (1.3)
No	153 (97.5)
Unknown	2 (1.3)
Total	157
**Other neonatal problem/abnormality**	
Yes	9 (5.7)
No	146 (93.0)
Unknown	2 (1.3)
Total	157

AE, adverse event; APGAR, Appearance, Pulse, Grimace, Activity, Respiration; AZCQ, azithromycin-chloroquine; SD, standard deviation.

*N displayed in the table was based on live born infants with either a date of birth or an AE record.

**Newborn without congenital malformations / anomalies and neonatal problems / abnormalities.

## Discussion

This open-label, out-patient study in five countries in sub-Saharan Africa was designed to assess parasitological cure rates up to 42 days following a single, 3-day dose of AZCQ given during the second or third trimester to pregnant women in their first or second pregnancy. In this study, a single, 3-day dose of AZCQ was well-tolerated and effective in reducing *P*. *falciparum* parasitemia in asymptomatic pregnant women with confirmed *P*. *falciparum* parasitemia.

There were no deaths in the mother group, and the occurrence of SAEs was low, with no serious TEAEs or severe TEAEs in the mother group up to day 35. The most frequent treatment-related TEAEs in the mother group were vomiting (20.2%) and dizziness (19.6%). After day 35, there were four neonate deaths due to SAEs, none of which were considered to be treatment-related.

An accompanying Phase 3 pivotal clinical trial (NCT01103063) was designed to assess AZCQ versus SP for IPTp in East and Southern sub-Saharan Africa. This trial was terminated based on results of a pre-planned interim analysis, which showed that the futility boundary for efficacy had been crossed.

Despite the failure of the pivotal Phase 3 trial, our findings showed that parasitological cure rates to a single, 3-day dose of AZCQ were high throughout the long duration (42 days) of the study, which was consistent with previous studies and further confirmed by sensitivity analyses in which study drop-outs were considered to be treatment failures.

The reasons why this AZCQ IPTp treatment regimen failed to demonstrate superiority versus SP in the Phase 3 pivotal trial are beyond the scope of this article, but clearly, the data we present here did not appear to suggest that AZCQ IPTp treatment would not be a viable treatment option. Another potential alternative to SP IPTp, DHA-P, was recently described [24, 25]. Interestingly, a recent study on mefloquine versus SP IPTp, showed that although mefloquine reduced the incidence of maternal parasitemia at the time of birth, there was no difference versus SP in the prevalence of LBW [26]. The investigators postulated that the improved malaria control strategies in place throughout the trial led to overall reductions in malaria transmission rate, thereby reducing the potential impact of malaria on birth outcomes relative to other confounding factors [26]. Perhaps, in large-scale Phase 3 clinical trials, the improvements in malaria control and access to health care that are provided to all participants, in either treatment group, reduce the influence of malaria on birth outcomes, thereby reducing the statistical power to detect any difference between treatment interventions.

## Supporting Information

S1 File TREND Checklist. (DOCX)

S2 File Study Protocol. (PDF)

## Abbreviations

AE: adverse event
AZCQ: azithromycin-chloroquine
CI: confidence interval
CV: coefficient of variation
DECQ: desethyl-CQ
IPTp: intermittent preventive treatment in pregnancy
ITT: intention to treat
LBW: low birth weight
MITT: modified intention to treat
PCR: polymerase chain reaction
PP: per protocol
SD: standard deviation
SP: sulfadoxine-pyrimethamine
TEAE: treatment emergent adverse event

## References

[1] Greenwood B, Mutabingwa T (2002) Malaria in 2002. Nature 415: 670–672. 10.1038/415670a 11832954

[2] Wright GJ, Rayner JC (2014) Plasmodium falciparum erythrocyte invasion: combining function with immune evasion. PLoS Pathog 10: e1003943 10.1371/journal.ppat.1003943 24651270 PMC3961354

[3] Laishram DD, Sutton PL, Nanda N, Sharma VL, Sobti RC, Carlton JM, et al (2012) The complexities of malaria disease manifestations with a focus on asymptomatic malaria. Malar J 11: 29 10.1186/1475-2875-11-29 22289302 PMC3342920

[4] World Health Organization (2013) World Malaria Report 2013.

[5] Menendez C (2006) Malaria during pregnancy. Curr Mol Med 6: 269–273. 16515517 10.2174/156652406776055186

[6] van Eijk AM, Ayisi JG, ter Kuile FO, Misore AO, Otieno JA, Rosen DH, et al (2002) Risk factors for malaria in pregnancy in an urban and peri-urban population in western Kenya. Trans R Soc Trop Med Hyg 96: 586–592. 12625128 10.1016/s0035-9203(02)90319-6

[7] Rijken MJ, McGready R, Boel ME, Poespoprodjo R, Singh N, Syafruddin D, et al (2012) Malaria in pregnancy in the Asia-Pacific region. Lancet Infect Dis 12: 75–88. 10.1016/S1473-3099(11)70315-2 22192132

[8] Dellicour S, Tatem AJ, Guerra CA, Snow RW, ter Kuile FO (2010) Quantifying the number of pregnancies at risk of malaria in 2007: a demographic study. PLoS Med 7: e1000221 10.1371/journal.pmed.1000221 20126256 PMC2811150

[9] Chico RM, Pittrof R, Greenwood B, Chandramohan D (2008) Azithromycin-chloroquine and the intermittent preventive treatment of malaria in pregnancy. Malar J 7: 255 10.1186/1475-2875-7-255 19087267 PMC2632633

[10] World Health Organization Organization (2007) Malaria in pregnancy Guidelines for measuring key monitoring and evaluation indicators. France: WHO.

[11] World Health Organization Organization (2012) Updated WHO policy recommendation. Intermittent Preventive Treatment of malaria in pregnancy using Sulfadoxine-Pyrimethamine (IPTp-SP).

[12] Geiger C, Compaore G, Coulibaly B, Sie A, Dittmer M, Sanchez C, et al (2014) Substantial increase in mutations in the genes pfdhfr and pfdhps puts sulphadoxine-pyrimethamine-based intermittent preventive treatment for malaria at risk in Burkina Faso. Trop Med Int Health 19: 690–697. 10.1111/tmi.12305 24674355

[13] ter Kuile FO, van Eijk AM, Filler SJ (2007) Effect of sulfadoxine-pyrimethamine resistance on the efficacy of intermittent preventive therapy for malaria control during pregnancy: a systematic review. JAMA 297: 2603–2616. 10.1001/jama.297.23.2603 17579229

[14] Spalding MD, Eyase FL, Akala HM, Bedno SA, Prigge ST, Coldren RL, et al (2010) Increased prevalence of the pfdhfr/phdhps quintuple mutant and rapid emergence of pfdhps resistance mutations at codons 581 and 613 in Kisumu, Kenya. Malar J 9: 338 10.1186/1475-2875-9-338 21106088 PMC3001743

[15] Karema C, Imwong M, Fanello CI, Stepniewska K, Uwimana A, Nakeesathit S, et al (2010) Molecular correlates of high-level antifolate resistance in Rwandan children with Plasmodium falciparum malaria. Antimicrob Agents Chemother 54: 477–483. 10.1128/AAC.00498-09 19841150 PMC2798539

[16] Chico RM, Chandramohan D (2011) Azithromycin plus chloroquine: combination therapy for protection against malaria and sexually transmitted infections in pregnancy. Expert Opin Drug Metab Toxicol 7: 1153–1167. 10.1517/17425255.2011.598506 21736423 PMC3170143

[17] Ohrt C, Willingmyre GD, Lee P, Knirsch C, Milhous W (2002) Assessment of azithromycin in combination with other antimalarial drugs against Plasmodium falciparum in vitro. Antimicrob Agents Chemother 46: 2518–2524. 10.1128/AAC.46.8.2518-2524.2002 12121927 PMC127390

[18] Pereira MR, Henrich PP, Sidhu AB, Johnson D, Hardink J, Van Deusen J, et al (2011) In vivo and in vitro antimalarial properties of azithromycin-chloroquine combinations that include the resistance reversal agent amlodipine. Antimicrob Agents Chemother 55: 3115–3124. 10.1128/AAC.01566-10 21464242 PMC3122405

[19] Sagara I, Oduro AR, Mulenga M, Dieng Y, Ogutu B, Tiono AB, et al (2014) Efficacy and safety of a combination of azithromycin and chloroquine for the treatment of uncomplicated Plasmodium falciparum malaria in two multi-country randomised clinical trials in African adults. Malar J 13: 458 10.1186/1475-2875-13-458 25425434 PMC4364337

[20] Chandra R, Ansah P, Sagara I, Sie A, Tiono AB, Djimdé AA, et al (2015) Comparison of azithromycin plus chloroquine versus artemether-lumefantrine for the treatment of uncomplicated Plasmodium falciparum malaria in children in Africa: a randomized, open-label study. Malar J 14: 108 10.1186/s12936-015-0620-8 25881046 PMC4358906

[21] World Health Organization Organization (2003) Assessment and monitoring of antimalarial drug efficacy for the treatment of uncomplicated falciparum malaria.

[22] Zhao Q, Purohit V, Cai J, RR L, Chandra R (2013) Relative bioavailability of a fixed-combination tablet formulation of azithromycin and chloroquine in healthy adult subjects. J Bioequiv Availab 5: 1–5.

[23] Djimdé A, Doumbo OK, Cortese JF, Kayentao K, Doumbo S, Diourté Y, et al (2001) A molecular marker for chloroquine-resistant falciparum malaria. N Engl J Med 344: 257–263. 10.1056/NEJM200101253440403 11172152

[24] Desai M, Gutman J, L'lanziva A, Otieno K, Juma E, Kariuki S, et al (2015) Intermittent screening and treatment or intermittent preventive treatment with dihydroartemisinin-piperaquine versus intermittent preventive treatment with sulfadoxine-pyrimethamine for the control of malaria during pregnancy in western Kenya: an open-label, three-group, randomized controlled superiority trial. Lancet 386:2507–19. 10.1016/S0140-6736(15)00310-4 26429700 PMC4718402

[25] Kakuru A, Jagannathan P, Muhindo MK, Natureeba P, Awori P, Nakalembe M, et al (2016) Dihydroartemisinin-piperaquine for the prevention of malaria in pregnancy. N Engl J Med 374:928–39. 10.1056/NEJMoa1509150 26962728 PMC4847718

[26] Gonzalez R, Desai M, Macete E, Ouma P, Kakolwa MA, Abdulla S, et al (2014) Intermittent Preventive Treatment of Malaria in Pregnancy with Mefloquine in HIV-Infected Women Receiving Cotrimoxazole Prophylaxis: A Multicenter Randomized Placebo-Controlled Trial. PLoS Med 11: e1001735 10.1371/journal.pmed.1001735 25247995 PMC4172537

