# Supplementary material for: Parasitological Clearance Rates and Drug Concentrations of a Fixed Dose Combination of Azithromycin-Chloroquine in Asymptomatic Pregnant Women with Plasmodium Falciparum Parasitemia: An Open-Label, Non-Comparative Study in Sub-Saharan Africa
Source: PLoS One. 2016 Nov 18;11(11):e0165692. doi: 10.1371/journal.pone.0165692 (PMC5115659; doi:10.1371/journal.pone.0165692)
Supplement: S2 File — (PDF) [file pone.0165692.s002.pdf]

CLINICAL PROTOCOL

AN OPEN LABEL, NON-COMPARATIVE STUDY TO EVALUATE PARASITOLOGICAL CLEARANCE RATES AND PHARMACOKINETICS OF AZITHROMYCIN AND CHLOROQUINE FOLLOWING ADMINISTRATION OF A FIXED DOSE COMBINATION OF AZITHROMYCIN AND CHLOROQUINE (AZCQ) IN ASYMPTOMATIC PREGNANT WOMEN WITH PLASMODIUM FALCIPARUM PARASITEMIA IN SUB-SAHARAN AFRICA

**Compound:**	Azithromycin Dihydrate and Chloroquine Phosphate
**Compound Name (if applicable):**	Azithromycin/Chloroquine Combination
**US IND Number (if applicable):**	N/A
**Protocol Number:**	A0661201
**Phase:**	Phase 3
**Version and Date:**	Final<br>14 February 2010

This document contains confidential information belonging to Pfizer. Except as otherwise agreed to in writing, by accepting or reviewing this document, you agree to hold this information in confidence and not copy or disclose it to others (except where required by applicable law) or use it for unauthorized purposes. In the event of any actual or suspected breach of this obligation, Pfizer must be promptly notified.

090177e1811d3ccb\Approved\Approved On: 19-Feb-2010 20:59

090177e1811d3ccb\Approved\Approved On: 19-Feb-2010 20:59

PROTOCOL SUMMARY

Indication

Intermittent preventive treatment of *falciparum* malaria in pregnant women (IPTp).

Background and Rationale

Malaria is a serious protozoal infection caused by any of the five species of *Plasmodia* and transmitted by anopheline mosquitoes; it continues to be one of the largest global health problems.[1 2] As per the World Health Organization (WHO) *World Malaria Report* 2009,[3] about half of the world's population is at risk of infection in 108 endemic countries and territories. In 2008, there were an estimated 243 million cases and 863,000 malaria-related deaths.[3] Malaria in pregnancy (MIP) is one of the most common causes of preventable mortality and morbidity in pregnant women and infants in sub-Saharan Africa. An estimated 30 million pregnancies are at risk of malaria infection in sub-Saharan Africa each year;[4] about 200,000 infants and 10,000 women die of malaria in pregnancy each year.[5]

Important progress has been made in the control of malaria in pregnancy in Africa with the introduction of intermittent preventive treatment in pregnancy (IPTp). The WHO recommends the administration of IPTp with sulfadoxine-pyrimethamine (SP) during antenatal visits in high malaria transmission areas of sub-Saharan Africa.[6] However, SP resistance has become widespread especially in East and Southern Africa, greatly limiting the protective effect of IPTp with SP. The development of safe, efficacious and affordable replacement of SP for IPTp is an urgent priority.

The combination of azithromycin (AZ) and chloroquine (CQ) could potentially replace SP for IPTp. AZ and CQ are synergistic in vitro and in vivo against CQ resistant strains of *P. falciparum*. Co-administration of AZ and CQ has demonstrated efficacy, safety and tolerability in two multi-country Phase 3 clinical studies (A0661134 and A0661155) in the treatment of symptomatic uncomplicated malaria in non-pregnant adults in sub-Saharan Africa. AZ and CQ have been on the market for several years and have extensive safety records in adults, children and pregnant women. Both AZ and CQ have been widely used in all trimesters of pregnancy and are considered safe in pregnant women as individual agents. In high-income countries, AZ is commonly used to treat and prevent sexually transmitted infections (STIs) including *Neisseria gonorrhoeae* and *Chlamydia trachomatis* infections. In sub-Saharan Africa, two randomized controlled clinical trials.[21, 22] found that a single dose of 1000 mg AZ was comparable to benzathine penicillin G in curing syphilis. The incidence of adverse fetal outcomes has been shown to be reduced by about 30% when these three STIs were treated in pregnancy.[23] In addition, AZ has also demonstrated protective effect against *Trichomonas vaginalis* when used as chemoprophylaxis[24] and reduced the risk of preterm delivery attributable to *T. vaginalis*, even in the second trimester when first-line treatment, metronidazole, should be avoided.[25]

A fixed dose combination tablet formulation of AZ and CQ (AZCQ, 250 mg AZ/155 mg CQ base) has been developed specifically for the IPTp indication and will be evaluated in IPTp studies. In a clinical pharmacology study A0661186, AZCQ administration resulted in the systemic exposures to AZ, CQ and des-ethyl CQ (an active metabolite of CQ) at levels comparable to those achieved following administration of commercial standalone tablets of AZ (Zithromax) and CQ (Aralen) evaluated in adult treatment studies.

A Phase 3 randomized controlled clinical study to demonstrate the superiority of AZCQ IPTp regimen over SP IPTp regimen is planned in East and Southern sub-Saharan Africa where SP resistance is emerging as an issue and where SP is the current standard of care for IPTp. This will be the pivotal study for regulatory submissions to European Medicines Agency (EMA) and to the national regulatory agencies in Sub-Saharan African countries.

AZCQ is expected to exert IPTp effect through multiple mechanisms including peripheral parasitological clearance in pregnant women, prevention of re-infections, prevention/treatment of placental malaria and prevention/treatment of STIs. The relative contributions of these mechanisms towards IPTp effect are not yet known. The study A0661201 is an open label, non-comparative parasitological clearance study of a single 3-day treatment course of AZCQ in about 166 pregnant women in second and third trimesters of pregnancy with asymptomatic parasitemia. This study will help to characterize the magnitude of parasitological clearance required for the IPTp effect observed in A0661158 study, and will serve as a supportive study in IPTp regulatory dossier. A0661201 study will also evaluate pharmacokinetic (PK) exposures, of both AZ and CQ in pregnant women following administration of a single 3-day treatment course of AZCQ. Subjects will be followed up to Day 42 after the first dose, and followed through delivery or to pregnancy termination for safety assessments of Exposure in Utero (EIU).

Objectives

The primary objective is to evaluate the peripheral parasitological clearance rate of AZCQ on Day 28 polymerase chain-reaction (PCR) corrected following the first dose of 3-day dosing regimen of AZCQ in asymptomatic pregnant women with *P. falciparum* parasitemia.

Secondary objectives include the evaluation of:

1. Parasitological clearance rates (PCR corrected) at Days 7, 14, 21, 35, and 42 post first dose of study medication;

2. Parasitological clearance rates (PCR uncorrected) at Days 7, 14, 21, 28, 35, and 42 post first dose of study medication;

3. Pharmacokinetic exposure of AZCQ;

4. Safety and tolerability of AZCQ.

090177e1811d3ccb\Approved\Approved On: 19-Feb-2010 20:59

090177e1811d3ccb\Approved\Approved On: 19-Feb-2010 20:59

Study Design

This is a Phase 3, open label, single arm non-comparative out-patient study in pregnant women during their second and third trimesters of pregnancy. Women will be screened for peripheral parasitaemia. Women who are screened but not included in the study (screen failures) will be referred to their doctors and will be given standard antenatal care (ANC) including IPTp. Subjects with asymptomatic parasitemia (counts of 80 -100,000/ μL) will receive a single 3-day course of AZCQ IPTp regimen. They will be followed up on a weekly basis up to Day 42, and following delivery or at termination of pregnancy for EIU safety assessments. After completing Day 42 evaluation, all subjects will continue to receive standard antenatal care including IPTp with SP if the gestational age allows additional IPTp course(s). The peripheral parasitological response will be evaluated on Days 7, 14, 21, 28, 35 and 42. Pharmacokinetics evaluation will be conducted on blood samples collected from the subjects who consented for such test. Systemic concentrations of AZ, CQ and desethyl-CQ (active metabolite of CQ) will be evaluated on Day 0 predose, Day 2 predose, 2 hours (as close to 2 hours as possible) and 8 hours (window: 4 to 12 hours) post dose, and at a random time point on Days 7 and 14. In addition, due to the long half-life of CQ, systemic concentrations of CQ and desethyl-CQ will also be measured at a random time point on Days 21 and 28. All subjects will be followed up for pregnancy outcomes as a safety endpoint. Long lasting insecticidal-treated bednets (LLITs) will be given to all subjects on Day 0 of the study with the instructions to use them; installation of LLINs will be verified during the Day 1 home visit by fieldworker(s).

Study Treatment

Study drug is a fixed dose tablet of AZCQ containing 250 mg AZ and 155 mg CQ base. All subjects will be administered a single 3-day course of AZCQ IPTp regimen: a single dose of 1000 mg AZ/620 mg CQ base (4 fixed dose combination tablets of AZCQ: 250mg/155mg) administered *per os* (PO, orally) once daily for 3 days (Days 0, 1, 2). The first dose (on Day 0) and the third dose (on Day 2) will be administered under supervision by investigators at the antenatal care (ANC) facility, and the second dose on Day 1 will be administered under supervision by a field worker at home.

Main Endpoints

<u>Primary Efficacy Endpoint</u>

- Parasitological response (PCR corrected) at Day 28 post first dose of study medication.

<u>Secondary Parasitological Clearance Endpoints</u>

- Parasitological responses (PCR corrected) at Days 7, 14, 21, 35, and 42 post first dose of study medication;

- Parasitological responses (PCR uncorrected) at Days 7, 14, 21, 28, 35, and 42 post first dose of study medication;

- Parasite counts at each visit – number of asexual *P. falciparum* parasites per microliter of blood.

Pharmacokinetic Evaluation

- Pharmacokinetic exposure of AZCQ.

Safety Endpoints

- Safety and tolerability endpoints include spontaneously reported adverse events, temperature, hemoglobin concentrations, and the EIU safety assessment.

Statistical Methods

This study is designed to estimate the incidence for the primary endpoint. No statistical hypothesis will be tested.

The proportion of subjects with parasitological response (PCR corrected) at Day 28 following the first dose of study medication will be estimated for the primary endpoint using the modified intent-to-treat (MITT) and per protocol (PP) subject populations. Similar analysis will also be performed for an intent-to-treat (ITT) population will also be defined. The proportion will be estimated from the Kaplan-Meier curve of the time to the first occurrence of parasitological failure (PCR corrected). Use of the Kaplan-Meier curve/product limit estimator is recommended by WHO[8] and has been used in analyzing primary efficacy endpoints in late stage antimalarial treatment clinical trials. See Section 9 for details.

An early analysis of the data will be performed on parasitological clearance endpoints including the primary endpoint, when all subjects complete Day 42 visit (ie, 42 days of follow-up post first dose), or withdraw from the study early.

Number of Subjects

Approximately 166 subjects will be enrolled in the study. Since this is a supportive study to understand one of the possible mechanisms for IPTp effect, it is not powered for formal hypothesis testing. The sample size will yield a half width of a 2-sided 95% confidence interval (CI) to be ≤5 percentage points with probability=0.80 for the primary efficacy endpoint, using a large sample normal approximation to the binomial, provided the true underlying incidence is ≥95%, and assuming approximately 10% will drop out.

Independent Data Monitoring Committee (IDMC)

The IDMC constituted for IPTp will review safety data at regular intervals to be determined at the time of DMC charter development.

090177e1811d3ccb\Approved\Approved On: 19-Feb-2010 20:59

Study Schematic

Baseline/Day 0

Pregnant women age between ≥16 and ≤35 years old
AND
Primigravidae and secundigravidae at ≥14 and ≤30 weeks, single pregnancy with gestational age confirmed by ultrasound examination
AND
Asymptomatic parasitemia with blood smears positive for Plasmodium falciparum (counts of 80 -100,000/ µL)
AND
Hemoglobin ≥8 g/dL
AND
No use of antimalarial drugs in previous 4 weeks
AND
A personally signed and dated informed consent document, and assent as appropriate

TREATMENT

The first dose on Day 0 and the third dose on Day 2 are administered under supervision during the ANC visit. The second dose on Day 1 will be administered at home by the fieldworkers.

AZCQ Treatment
1000 mg AZ and 620 mg base of CQ
(4 fixed dose combination tablets of AZCQ: 250mg/155mg)
PO once daily for 3 days

Days 0, 1, 2

Follow-up VISITS
At Days 7, 14, 21, 28, 35, 42

Safety Follow-up VISITS
for Exposure in Utero Assessment
Following Delivery or Pregnancy Termination
at or post Delivery

090177e1811d3ccb\Approved\Approved On: 19-Feb-2010 20:59

090177e1811d3ccb\Approved\Approved On: 19-Feb-2010 20:59

SCHEDULE OF ACTIVITIES

Protocol Activity	Treatment			Follow Up Visits						
	Day 0[a]	Day 1	Day 2	Day 7	Day 14	Day 21	Day 28	Day 35	Day 42	At/Post Delivery[b]
Informed Consent Process	X									
Medical History	X									
Obstetrical History	X									
Concomitant Treatment History	X	X	X	X	X	X	X	X	X	X
Rapid Diagnostic Test (eg, Binax)	X									
Complete Physical Exam	X									
Limited Physical Exam									X	
Oral Temperature	X	X	X	X	X	X	X	X	X	
Routine Obstetric checkup	X									
Ultrasound[c]	X									
Laboratory										
Peripheral Blood Smears[d]	X			X[d]	X[d]	X[d]	X[d]	X[d]	X[d]	
Hemoglobin (Hb)	X								X	
Urine Pregnancy Test[e]	X									
Urine Test for Glucose & Protein	X									
Blood Blot for Molecular Testing										
Molecular Genotyping for *P. Falciparum*[f]	X			X[f]	X[f]	X[f]	X[f]	X[f]	X[f]	
Genetic Marker for CQ Resistance	X									
Administer Treatment Azithromycin-Chloroquine (AZCQ[g])	X	X	X							
Distribute Long lasting insecticidal-treated bednets (LLITs)	X									
Confirm the Installation of LLINs at Home by Fieldworkers		X								
Blood Samples for AZ, CQ and desethyl-CQ Analysis[h]	X[i]		X[j]	X[k]	X[k]	X[k]	X[k]			
Adverse Events Assessment	X	X	X	X	X	X	X	X	X	
Exposure in Utero (EIU) Assessment[l]										X

090177e1811d3ccb\Approved\Approved On: 19-Feb-2010 20:59

[a] All tasks should be performed prior to the first dose except AE assessment and bednet distribution.
[b] All subjects should be followed up following delivery or at termination of pregnancy for EIU assessment. In case of no associated serious adverse events (SAE), the follow-up visit should take place within 14 calendar days after expected date of delivery or termination of pregnancy.
[c] Ultrasound will be performed only if the urine pregnancy test is positive.
[d] Peripheral blood smears (thick and thin) will be prepared at visits on Day 0 (predose) and weekly intervals afterwards. Two smears per subject at each time point.
[e] If not done at any prior ANC visit for this pregnancy.
[f] The blood blot will be collected at baseline (predose) and each visit through Day 42 but evaluated only if positive by the blood smear following initial clearance.
[g] The first dose on Day 0 and the third dose on Day2 will be administered under supervision by investigator at the antenatal care (ANC) clinic, and the second dose on Day 1 will be administered at home under supervision of a field worker(s).
[h] PK evaluation will be conducted on blood samples collected from the subjects who consented for such test. Insert intravenous catheter as deemed necessary. Subjects who received even one dose will be followed up for all subsequent PK sample collection. The exact date and time of blood sample collection will be recorded.
[i] Two PK blood specimens (one for AZ and one for CQ and desethyl-CQ analyses) will be collected at predose.
[j] Two PK blood specimens (one for AZ and one for CQ and desethyl-CQ analyses) will be collected at predose, 2 hours (as close to 2 hours as possible) and 8 hours (window: 4 to 12 hours) following dosing on Day 2.
[k] Two PK blood specimens (one for AZ and one for CQ and desethyl-CQ analyses) will be collected at visits on Days 7 and 14 (a random time point), and only blood sample (for CQ and desethyl-CQ analyse) will be collected on Days 21 and 28 samples (a random time point).
[l] EIU assessment: Every attempt should be made to follow up the subject following delivery or pregnancy termination. In case of no associated serious adverse events (SAE), subjects should be followed up by fieldworkers at clinic or at home within 14 calendar days after expected date of delivery or termination of pregnancy. EIU assessment includes maternal information, delivery and neonatal information. Fetal information will be collected in the event of pregnancy termination.

TABLE OF CONTENTS

1. INTRODUCTION 13
1.1. Indication 13
1.2. Background 13
1.3. Azithromycin 16
1.3.1. Non-Clinical Summary of Azithromycin 16
1.3.1.1. Pharmacology and Microbiology of Azithromycin 16
1.3.1.2. Non-clinical Activity of Azithromycin as an Antimalarial Agent 16
1.3.1.3. Non-clinical Safety of Azithromycin 17
1.3.2. Clinical Summary of Azithromycin 17
1.3.2.1. Clinical Pharmacokinetics of Azithromycin 17
1.3.2.2. Clinical Efficacy of Azithromycin in Malaria 19
1.3.2.3. Clinical Safety and Tolerability of Azithromycin 20
1.4. Chloroquine 21
1.4.1. Non-Clinical Summary of Chloroquine 21
1.4.1.1. Pharmacology and Microbiology of Chloroquine 21
1.4.1.2. Non-clinical Safety of Chloroquine 21
1.4.2. Clinical Summary of Chloroquine 22
1.4.2.1. Clinical Pharmacology of Chloroquine 22
1.4.2.2. Clinical Efficacy of Chloroquine Monotherapy in Malaria 22
1.4.2.3. Clinical Safety and Tolerability of Chloroquine 23
1.5. Azithromycin-Chloroquine Combination (AZCQ) 24
1.5.1. Non-Clinical Summary Information 24
1.5.1.1. Non-Clinical Efficacy of the Azithromycin and Chloroquine Combination against *P. falciparum* 24
1.5.1.2. Non-clinical Safety of Azithromycin and Chloroquine Combination 24
1.5.2. Clinical Summary Information for Azithromycin and Chloroquine Combination 25
1.5.2.1. Clinical Pharmacology of Azithromycin and Chloroquine Combination 25
1.5.2.2. Clinical Efficacy of Azithromycin and Chloroquine Combination against *P. falciparum* 26
1.5.2.3. Clinical Safety and Tolerability of Azithromycin and Chloroquine Combination 27
1.6. Rationale 28
1.6.1. Dosing Regimen Selection (AZCQ) 29
2. STUDY OBJECTIVES AND ENDPOINTS 30
2.1. Objectives 30
2.2. Endpoints 30
3. STUDY DESIGN 31

090177e1811d3ccb\Approved\Approved On: 19-Feb-2010 20:59

090177e1811d3ccb\Approved\Approved On: 19-Feb-2010 20:59

4. SUBJECT SELECTION 31
4.1. Inclusion Criteria 31
4.2. Exclusion Criteria 32
4.3. Randomization Criteria 33
4.4. Life Style Guidelines 33
5. STUDY TREATMENTS 34
5.1. Allocation to Treatment 34
5.2. Breaking the Blind 34
5.3. Drug Supplies 34
5.3.1. Formulation and Packaging 34
5.3.2. Preparation and Dispensing 34
5.3.3. Administration 35
5.3.4. Compliance 35
5.4. Drug Storage and Drug Accountability 35
5.5. Concomitant Medication(s) 36
6. STUDY PROCEDURES 36
6.1. Study Period 36
6.1.1. Baseline/Day 0 Visit (at ANC clinic/center) 36
6.1.2. Days 1 Home Visit (by the field worker) 37
6.1.3. Days 2 Visit (at ANC clinic/center) 38
6.1.4. Follow-up Visits on Days 7, 14, 21, 28, 35, 42 at ANC clinic/center) 38
6.1.5. Unscheduled Visits through Day 42 39
6.1.6. Safety Follow-up Visit for EIU Assessment 39
6.2. Subject Withdrawal 40
7. ASSESSMENTS 41
7.1. Parasite Clearance Assessments 41
7.2. Safety Assessments 41
7.3. Pharmacokinetic Assessments 42
7.3.1. Serum for Analysis of Azithromycin 43
7.3.2. Plasma for Analysis of Chloroquine and Desethyl-Chloroquine 43
7.3.3. Shipment of Pharmacokinetic Samples 44
8. ADVERSE EVENT REPORTING 44
8.1. Adverse Events 44
8.2. Reporting Period 44
8.3. Definition of an Adverse Event 45
8.4. Abnormal Test Findings 45
8.5. Serious Adverse Events 46
8.6. Hospitalization 46

8.7. Severity Assessment....48
8.8. Causality Assessment....48
8.9. Exposure During Pregnancy....48
8.10. Withdrawal Due to Adverse Events (See also section on Subject Withdrawal)....49
8.11. Eliciting Adverse Event Information....49
8.12. Reporting Requirements....50
8.12.1. Serious Adverse Event Reporting Requirements....50
8.12.2. Non-Serious Adverse Event Reporting Requirements....50
8.12.3. Medical Device Complaint Reporting Requirements....51
8.12.4. Sponsor Reporting Requirements to Regulatory Authorities....51
9. DATA ANALYSIS/STATISTICAL METHODS....51
9.1. Sample Size Determination....51
9.2. Parasitological Response Analysis....51
9.2.1. Analysis of Primary Endpoint....52
9.2.2. Analysis of Secondary Endpoints....53
9.3. Safety Analysis....53
9.4. Pharmacokinetic Analysis....54
9.5. Interim Analysis....54
9.6. Independent Data Monitoring Committee (IDMC)....54
10. QUALITY CONTROL AND QUALITY ASSURANCE....54
11. DATA HANDLING AND RECORD KEEPING....55
11.1. Case Report Forms / Electronic Data Record....55
11.2. Record Retention....55
12. ETHICS....56
12.1. Institutional Review Board (IRB)/Independent Ethics Committee (IEC)....56
12.2. Ethical Conduct of the Study....56
12.3. Subject Information and Consent....56
12.4. Reporting of safety issues and serious breaches of the protocol or ICH GCP....57
13. DEFINITION OF END OF TRIAL....57
13.1. End of Trial in all Participating Countries....57
14. SPONSOR DISCONTINUATION CRITERIA....57
15. PUBLICATION OF STUDY RESULTS....57
15.1. Communication of results by Pfizer....58
15.2. Publications by Investigators....59
16. REFERENCES....60

090177e1811d3ccb\Approved\Approved On: 19-Feb-2010 20:59

TABLES

Table 1. Azithromycin Pharmacokinetic Data....................18
Table 2. PK Parameters from A0661186 Study Formulation....................25
Table 3. Severity Assessment....................48

FIGURES

Figure 1. Predicted Azithromycin Serum Concentrations in Healthy Subjects and Pregnant women....................19

APPENDICES

Appendix 1. Rapid Diagnostic Testing for *P. falciparum*....................68

090177e1811d3ccb\Approved\Approved On: 19-Feb-2010 20:59

1. INTRODUCTION

1.1. Indication

Intermittent Preventive Treatment of *falciparum* malaria in pregnant women (IPTp).

1.2. Background

Malaria is a serious protozoal infection caused by any of the five species of *Plasmodium* and transmitted by anopheline mosquitoes. *Plasmodium falciparum* is one of the leading causes of deaths world-wide caused by a single causative agent.[1 2] As per the World Health Organization (WHO) *World Malaria Report* 2009,[3] about half of the world's population is at risk of infection in 108 endemic countries and territories. There were an estimated 243 million cases and 863,000 malaria-related deaths in 2008.[3] Africa has the largest number of people living in high-risk areas and the most the malaria deaths. Pregnant women are more prone to malaria than non-pregnant women, especially during their first and second pregnancies. Malaria in pregnancy (MIP) is one of the most common causes of preventable morbidity and mortality in pregnant women and infants in sub-Saharan Africa. An estimated 30 million pregnancies are at risk of malaria infection in the region each year;[4] about 200,000 infants and 10,000 women die of malaria in pregnancy each year.[5]

Intermittent Preventive Treatment in Pregnancy (IPTp) is one of the four key strategies recommended by the WHO's Global Malaria Program for malaria control in high transmission areas, where approximately 1 in 4 pregnant women are parasitemic at any given time, but remain mostly asymptomatic.[6] Placental infection is of particular concern to fetal development and can only be diagnosed post-partum by placental histological examination. IPTp is based on administration of a complete curative dosing regimen of an antimalarial medicine at periodic intervals during pregnancy regardless of whether or not pregnant women are infected with malaria. IPTp has two primary objectives: (1) to clear asymptomatic peripheral and placental parasitemia, and (2) to provide intermittent chemoprophylaxis against malaria infection during pregnancy.[4]

The WHO recommends use of IPTp in endemic areas of sub-Saharan Africa with two or three (in HIV infected pregnant women) courses of sulfadoxine and pyrimethamine (SP) after fetal quickening in second trimester with each course given no less than one month apart, and all prior to the last month of pregnancy.[6] SP regimen is started in second trimester. As an anti-metabolite, SP is not safe in first trimester of pregnancy. For women with HIV infection, at least three treatment courses are recommended. IPTp with SP has been shown to be effective in reducing maternal anemia, placental malaria and low birth weight neonates.[7] It is estimated that universal coverage with IPTp would reduce all-cause neonatal mortality by 32%.[4] Thirty-three African countries have officially adopted IPTp as national policy in their national malaria control programs. The benefits of IPTp are, however, compromised by increasing resistance of *Plasmodium falciparum* to SP especially in East and Southern Africa. The in vivo SP resistance rates have typically been evaluated in terms of treatment failures in clinical trials in children with uncomplicated infection[8] but therapeutic efficacy measured in sick children does not predict efficacy in IPTp with SP.

090177e1811d3ccb\Approved\Approved On: 19-Feb-2010 20:59

Acquired immunity in adults contributes to the efficacy of partially effective drugs. This protection, however, is not uniform and it is least efficacious among primigravidae who are most vulnerable to the detrimental effects of MIP. It is likely that as rates exceed an undetermined threshold of resistance, SP may fail to provide protection even in multigravidae and hence there is an urgent need to find new, safe and effective drugs or drug combination for IPTp.[4] Also, while SP still seems to work reasonably well in West Africa, evidence suggests that gene-flow will prompt the spread of SP resistance from east to West Africa.[9]

An ideal antimalarial drug or drug combination for IPTp needs to be safe, well tolerated, and efficacious in preventing the detrimental effects of malaria on the mother and the fetus, and, ideally, not used concurrently as the first line treatment for symptomatic malaria. Few clinical trials have attempted to evaluate alternatives to SP in IPTp. Malaria in Pregnancy Consortium is conducting a multi-country clinical trial in East and Southern Africa comparing the IPTp efficacy and safety of SP and mefloquine. Tolerability remains an issue for mefloquine, however, as evidenced by a recent IPTp study in Benin in which 78% ($P<0.001$) of women who received a single dose (15 mg/kg) experienced adverse events including vomiting, dizziness, tiredness, and nausea; one subject had severe neuropsychiatric symptoms.[10] Two retrospective studies found associations between mefloquine exposure and spontaneous abortion,[11] and stillbirth.[12] These results remain unexplained and have not been observed in other studies. In Ghana, the combination of SP and amodiaquine (600 mg on Days 0 and 1, 400 mg on Day 2) was recently evaluated for possible use in IPTp; adverse events, most commonly body pains and weakness, dizziness, vomiting and nausea, were observed among 89% ($P=0.001$) of women following the first course.[13] Similar observations of poor amodiaquine tolerability have been reported in other studies.[14, 15]

Other antimalarials that are being investigated for IPTp are SP combinations with azithromycin (AZ) and artesunate. While AZ has been used in all trimesters of pregnancy to treat a variety of infections, there is no evidence of synergy between SP and AZ against *P. falciparum*. Artemisinins are among the most effective and rapidly acting antimalarials to date, but their use in non-clinical studies has been associated with fetal resorptions and embryotoxicity in rodents and rabbits with narrow therapeutic margins when given early in pregnancy.[16] No such evidence in clinical trials among pregnant women has been reported so far. The WHO currently recommends use of artemisinins for treatment of uncomplicated infections in pregnant women only when efficacious alternatives are not available.[17] As for IPTp use, the short half-life of artemisinins may restrict their utility for chemoprophylaxis. Artesunate and AZ would not likely be an appropriate combination either. The combination has produced poor *in vivo* results.[18] This, however, is not unexpected since *in vitro* pharmacodynamic interaction studies with this combination have also shown that artesunate and AZ are antagonistic to each other.[19] Therefore, options for efficacious, safe and well-tolerated preventive alternatives for pregnant women are limited. The need to develop new therapies is urgent.[20]

The combination of AZ and chloroquine (CQ) could potentially replace SP for IPTp. AZ and CQ combination is synergistic against CQ resistant strains of *P. falciparum* as demonstrated both in vitro and in vivo. Co-administration of AZ and CQ has demonstrated efficacy, safety

090177e1811d3ccb\Approved\Approved On: 19-Feb-2010 20:59

and tolerability in two multi-country Phase 3 clinical studies (A0661134 and A0661155) in the treatment of symptomatic uncomplicated malaria in adults in sub-Saharan Africa.
AZ and CQ have been on the market for several years and have extensive safety records in adults, children and pregnant women. Both AZ and CQ have been widely used in all trimesters of pregnancy and are considered safe in pregnant women as individual agents.
AZ has been used to treat and prevent sexually transmitted infections (STIs) including *Neisseria gonorrhoeae* and *Chlamydia trachomatis* infections. In two randomized controlled clinical trials,[21, 22] a single dose of AZ had comparable efficacy to benzathine penicillin G in efficacy in treating early syphilis. The incidence of adverse fetal outcomes has been shown to have reduced by about 30% when these three STIs were treated in pregnancy.[23] Activity of AZ against these infections should provide additional benefit. In addition, AZ has also demonstrated protective effect against *Trichomonas vaginalis* when used as chemoprophylaxis[24] and reduced the risk of preterm delivery attributable to *T. vaginalis,* even in the second trimester when first-line treatment, metronidazole, should be avoided.[25]

A fixed dose combination tablet formulation of AZ and CQ, AZCQ (250 mg AZ/155 mg CQ base) has been developed specifically for the IPTp indication and will be evaluated in clinical studies for IPTp indication.

A Phase 3 randomized controlled clinical study (A0661158) to demonstrate the superiority of AZCQ IPTp regimen over SP IPTp regimen is planned in countries in East and Southern Africa where SP is the current standard of care for IPTp and resistance to SP is a considerable issue. The study will be conducted in asymptomatic pregnant subjects enrolled during second trimester of pregnancy, and about half of the subjects will be primigravidae and secundigravidae pregnant women. Each subject will receive three IPTp courses of AZCQ or SP during antenatal care (ANC) visits at 4 - 8 week intervals. Subjects will be followed up at delivery or soon thereafter (within 7 days), and on Day 28 (window: Day 28 - Day 42) post-delivery. This will be the pivotal study for regulatory submissions to EMA (European Medicines Agency) and to the national regulatory agencies in sub-Saharan African countries.

The study A0661201 is an open label, non-comparative parasitological clearance study of a single 3-day treatment course of AZCQ in about 166 pregnant women in second and third trimester with asymptomatic parasitemia. This will help determine the magnitude of parasitological clearance required for the IPTp effect observed in A0661158 study and will be used to support the regulatory submissions of AZCQ for IPTp in sub-Saharan Africa. This study will also evaluate pharmacokinetic exposures of AZ and CQ in pregnant women following administration of a single 3-day treatment course of AZCQ. All subjects will be followed up on a weekly basis up to Day 42 following first dose of AZCQ, and following delivery or at termination of pregnancy for Exposure in Utero (EIU) safety assessments.

090177e1811d3ccb\Approved\Approved On: 19-Feb-2010 20:59

1.3. Azithromycin

1.3.1. Non-Clinical Summary of Azithromycin

1.3.1.1. Pharmacology and Microbiology of Azithromycin

AZ is a slow-acting anti-malarial macrolide,[26] an analogue of erythromycin with a nitrogen atom inserted into the macrolide nucleus. This pharmacological change enables greater penetration of drug into macrophages, fibroblasts and polymorphonuclear neutrophils, and enhanced accumulation within acidified vacuoles, extending the 1.5-hour half-life of erythromycin to 68 hours for AZ.[27] Stable at gastric pH, AZ has an absolute bioavailability of 37% following oral administration.[28] In animal studies, it has been shown to accumulates in hepatic, renal, pulmonary and splenic tissue,[29] and gradually leaches into the bloodstream over a one-week period.[30] Mild renal dysfunction and mild-to-moderate hepatic dysfunction do not affect excretion significantly.

AZ has significant activity against respiratory pathogens, both extracellular (eg, *Streptococcus pneumoniae*, *Haemophilus influenzae*, and *Moraxella catarrhalis*) and intracellular (eg, *Chlamydia pneumoniae*, *Legionella pneumophila*); selected sexually transmitted infections (eg, *Chlamydia trachomatis*, *Neisseria gonorrhoeae, Treponema pallidum,* and *Haemophilus ducreyi*)[21, 22, 31] as well as other atypical organisms such as *Bartonella* spp., non-tuberculous mycobacteria, *Rhodococcus equi*, and rickettsiae are susceptible to AZ. AZ also exhibits anti-protozoal activity (eg, *Toxoplasma gondii*, *Giardia. intestinalis*, *Entamoeba histolytica*)[32-34] and anti-paraplasmic activity including *Babesia microti*[35] and *P. falciparum*.

1.3.1.2. Non-clinical Activity of Azithromycin as an Antimalarial Agent

In vitro studies have shown that AZ has a 50% inhibitory concentration (IC_{50}) similar to other antibiotics used against malaria. In an *in vitro P. falciparum* model, AZ did not appear to exhibit cross-resistance with aminoquinolines, artemisinin derivatives, or quinine in an in vitro *P. falciparum* model.[36] The *in vitro* anti-malarial activity of AZ increased 200-fold against *P. falciparum* isolates when incubated between 24 and 48 hours, while its IC_{50} values drop as low as 35 nanomolar.[37] At 48-hours, AZ was 10-fold more active than erythromycin against CQ-resistant *P. falciparum*; the two compounds were equipotent, however, when CQ-sensitive parasites were exposed to the same drug concentration.[38, 39] Yeo and Rieckmann demonstrated that the minimum inhibitory concentrations (MICs) of AZ for both CQ-resistant and sensitive strains decreased over 48 to 96 hours and concluded that it behaved like other antibiotics in its slow rate of action against *P. falciparum*.[40] In India, AZ was tested *in vitro* against 10 strains of *P. falciparum* (5 CQ-resistant and 5 CQ-sensitive) and completely inhibited parasite growth in all 10 isolates at the highest concentration (40 μg/ml).[41]

090177e1811d3ccb\Approved\Approved On: 19-Feb-2010 20:59

In vivo animal studies lend further support to the role of AZ as an antimalarial agent. In the *P. berghei* murine model, AZ was shown to be more active than erythromycin with lower IC50s against both CQ-resistant and sensitive strains.[39] All mice were cured of parasitemia within three days after receiving a dose of AZ that achieved an IC_{50}. AZ was also more active than roxithromycin, clarithromycin, erythromycin and doxycycline in another study using a rodent model with *P. berghei*.[18]

1.3.1.3. Non-clinical Safety of Azithromycin

The non-clinical data for AZ suggest little if any evidence of toxicity. The non-clinical toxicology program included single-dose studies in rats, mice, and dogs, multiple-dose studies of up to 6 months in rats and dogs, fertility and reproductive developmental studies in rats, mice, or rabbits, neonatal studies in rats and dogs, and mechanistic studies in rats and dogs to characterize the extent, reversibility, and consequences of phospholipidosis. Genetic toxicology studies for AZ were also conducted. AZ did not produce toxicity in neonatal rats and dogs at the highest doses ranging from 60 to 140 mg/kg/day at approximately 5-10 times higher than human treatment dose. Phospholipidosis was observed in tissues of neonatal animals as was observed in adult animals. The clinical significance of this finding is unknown.

1.3.1.3.1. Non-clinical Safety of Azithromycin on Reproductive Systems

In fertility/reproductive non-clinical studies, AZ showed no evidence of producing fetal abnormalities in mice, rats, and rabbits at the highest doses ranging from 40 to 200 mg/kg/day. Fetal abnormalities or evidence of embryotoxicity were not observed in mice and rats dosed with AZ at 10, 20, 40, 50, 100, and 200 mg/kg/day (mice dosed on gestation Days 6-13, rats on Days 6-15). A slight delay in fetal ossification was noted when a decrease in maternal body weight occurred at doses of 100 and 200 mg/kg/day at approximately 7-15 times higher than human treatment dose. No evidence of impaired fertility due to AZ was found in rats at 10mg/kg/day. Rabbits dosed with AZ at 10, 20, and 40 mg/kg/day (on gestation Days 6-18) were not observed with fetal abnormalities or embryotoxicity. There was no evidence of teratogenicity in animal models at four-times the human treatment dose.[42-44]

1.3.2. Clinical Summary of Azithromycin

1.3.2.1. Clinical Pharmacokinetics of Azithromycin

The pharmacokinetics of AZ has been evaluated in several clinical pharmacology studies. Following oral administration of AZ tablets to healthy subjects, peak serum concentrations are achieved between 2 to 3 hours. AZ is widely distributed throughout the body with markedly higher and sustained exposure in tissues than in systemic circulation (up to 100 times of corresponding plasma or serum concentration) indicating that AZ is heavily tissue bound (per AZ label). The observed mean serum elimination half-life of AZ closely reflects the tissue depletion half-life of 2 to 3 days.[95, 96] The observed exposures of AZ and CQ in patients with malaria are consistent with that from healthy subjects.

090177e1811d3ccb\Approved\Approved On: 19-Feb-2010 20:59

Available AZ PK data suggests that no dosage adjustment is necessary in pregnant women. Pharmacokinetics of AZ following a single oral dose at 1000 mg was evaluated in 20 full term gravid pregnant women in the USA by Ramsey et al.[45] In this study, peak maternal serum AZ concentrations occurred within six hours following oral administration. AZ levels in myometrial, adipose, and placental tissues were much higher than serum concentrations. Although only 2.6% of a maternal dose perfuses the placenta,[46] placental AZ concentrations were maintained 6-15 times of the corresponding serum AZ concentrations during the first 72 hours post-dose (Table 1 below).[45]

Table 1. Azithromycin Pharmacokinetic Data

Specimen	Time After Azithromycin Administration (ng/mL)				
	6 h (n=2)	12 h (n=7)	24 h (n=5)	72 h (n=5)	168 h (n=1)
Maternal serum	311 ± 170	144 ± 79	63 ± 37	60 ± 31	<10.4
Maternal myometrium	1382 ± 549	1792 ± 1107	1299 ± 466	961 ± 419	36 ± 0
Maternal adipose tissue	1041 ± 706	494 ± 204	599 ± 330	551 ± 129	41 ± 0
Placenta	2130 ± 340	1196 ± 224	936 ± 223	915 ± 628	40 ± 0

Data are given as mean ± SD.

In a PK modeling analysis, AZ concentrations were predicted for pregnant women based on the known AZ exposures in full-term gravid pregnant women reported by Ramsey *et al*[45] and in healthy subjects described by Liu *et al.*[47] Predicted serum AZ concentrations in pregnant women were overlaid with the mean serum concentrations (with 95%CI) in healthy subjects (Figure 1), producing considerable overlap between the range of expected concentrations in both groups. Thus AZ exposures in pregnant women should be similar to healthy subjects.

090177e1811d3ccb\Approved\Approved On: 19-Feb-2010 20:59

Figure 1. Predicted Azithromycin Serum Concentrations in Healthy Subjects and Pregnant women

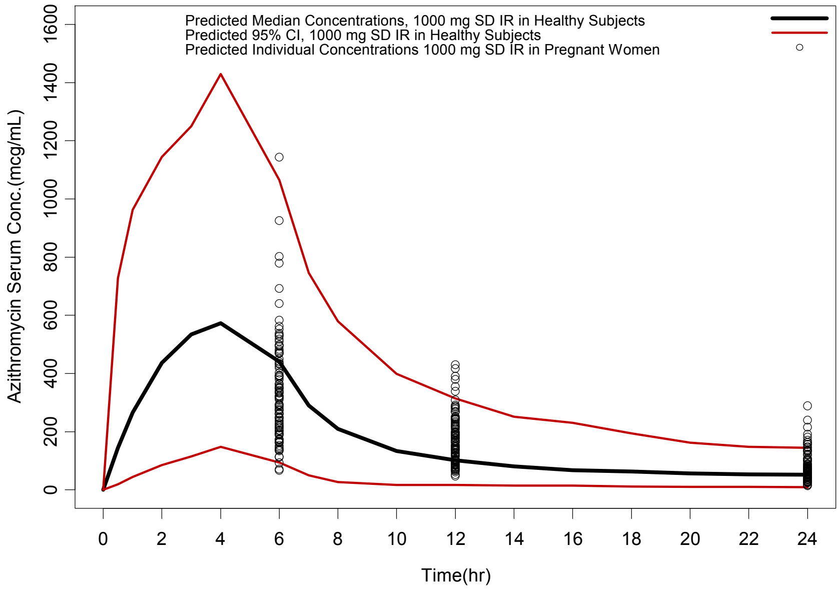

1.3.2.2. Clinical Efficacy of Azithromycin in Malaria

Daily regimens of 250 mg of AZ with a loading dose of 500 or 750 mg have shown an impressive chemoprophylactic effect against *P. vivax*. AZ had a 99% protective effect (95% CI, 93% to 100%) among semi-immune subjects in Indonesia over a 20-week period.[48] A similar protective efficacy, 98% (95% CI, 88% to 100%), was seen in Thailand[49] in a semi-immune population. By comparison, the chemoprophylactic effect of AZ against *P. falciparum* has been less impressive. Among semi-immune populations, an equivalent or higher loading dose with the same daily regimen resulted in protective efficacies of 83% (95% CI, 69% to 91%) in Kenya,[50] 71% (95% CI, -14% to 94%) in Thailand,[49] and 72% (95% CI, 50% to 84%) in Indonesia.[48]

Human challenge studies with one *P. falciparum* strain revealed only partial causal prophylaxis but 100% suppressive prophylaxis.[51, 52] Although this high level of protection has not been replicated in the field where multiple infections may be expected, these data suggested that AZ has the potential to be an effective, well-tolerated clinical prophylactic agent for *P. falciparum* malaria.

In vivo treatment efficacy of AZ was measured indirectly as part of a clinical trial of trachoma control in The Gambia. A retrospective analysis of children receiving oral AZ versus tetracycline eye ointment for trachoma revealed that symptomatic parasitemia and spleen indices were significantly lower in those who received AZ. These data were encouraging and prompted prospective studies evaluating the use of AZ in the treatment of

090177e1811d3ccb\Approved\Approved On: 19-Feb-2010 20:59

uncomplicated *falciparum* malaria.[19] In a phase 2 treatment trial in India (066-191), 1000 mg of AZ were given to patients with peripheral smears positive for *P. falciparum* or *P. vivax* once a day for three days. The analysis indicated suboptimal efficacy (33% at Day 28) with AZ monotherapy.[53]

1.3.2.3. Clinical Safety and Tolerability of Azithromycin

Doses of AZ between 500 mg and 2,000 mg have been used in all trimesters of human pregnancy for the treatment of upper and lower respiratory tract infections, skin diseases, *C. trachomatis*, mycoplasma and group B streptococci infections among women allergic to other antibiotics.[4]

Adults treated with a 1,000 mg oral dose of AZ report mild to moderate side-effects including diarrhea or loose stools (7%), nausea (5%), vomiting (2%), and vaginitis (2%); up to 1% of adults experience dizziness, headache, vertigo, and somnolence.[54]

1.3.2.3.1. Clinical Safety of Azithromycin in Pregnancy

Meta-analysis of eight randomized clinical trials among pregnant women with *C. trachomatis* infection found that AZ was associated with fewer gastrointestinal adverse events than erythromycin (OR = 0.11, 95% CI 0.07–0.18) and fewer total adverse events (OR = 0.11, 95% CI 0.07–0.18).[55]

Bar-Oz and colleagues conducted a prospective multi-center study of pregnancy outcomes between pregnant women exposed to one of the new macrolides (clarithromycin, roxithromycin and AZ) during the first trimester of pregnancy (n=161) and two comparison groups exposed to either other antibiotics or other non-teratogens (n=953). The rate of major malformations in the macrolide exposed group was 4.1% compared to 2.1% in the other antibiotic exposed group and 3.0% in the other non-teratogens group. The calculated odds ratio (OR) comparing macrolides to other non-teratogens was 1.41 (95 % confidence interval: 0.47-4.23). However, among the malformations in the macrolide group (4/97) only one case involved AZ (1/27) and the remainder involved roxithromcyin (3/31). Based on these data, AZ did not appear to confer a higher risk of malformations compared to other antibiotics and other non-teratogens.[56]

Cooper and colleagues conducted a retrospective cohort study of 30,049 infants from Tennessee Medicaid who had been exposed to fetal exposure to AZ and other antibiotics in utero compared to infants with no exposure to antibiotics (24521) in utero. Overall, 2.95% of the cohort had a confirmed major congenital malformation with major malformations ranging from 2.5% to 3.0% according to antibiotic groups (doxycycline 2.5%, erythromycin 2.6%, ciprofloxacin 2.8%, multiple 2.8%, AZ 2.9%, amoxicillin 2.9%, none 3.0%). Compared to infants with no exposure to antibiotics, there was no statistically significant increased risk of overall congenital malformations in the group exposed to AZ over the entire pregnancy (relative risk (RR) 1.00, 95% confidence interval 0.71, 1.42).[57]

090177e1811d3ccb\Approved\Approved On: 19-Feb-2010 20:59

Kalliani and colleagues conducted a randomized controlled pilot trial of SP alone, SP plus AZ and SP plus artesunate in the treatment of malaria in 141 pregnant women.[58] No significant differences in birth outcomes were noted between the three groups. Congenital anomalies were not examined in this study.

1.4. Chloroquine

1.4.1. Non-Clinical Summary of Chloroquine

1.4.1.1. Pharmacology and Microbiology of Chloroquine

CQ has been the first-line treatment of malaria in much of the world for most of the past 60 years. Due to widespread resistance, CQ is no longer recommended for treatment of *falciparum* malaria. Resistance to CQ is associated with polymorphisms in the *P. falciparum* food vacuole transporter protein (*pfcrt*) located on chromosome 7.[59] All *pfcrt* alleles from CQ-resistant strains, regardless of geographic origin, encode a conserved K76T amino acid substitution. The effect of *pfcrt* on CQ pharmacokinetics remains disputed. Some researchers have theorized that *pfcrt* enables protonated CQ to escape the food vacuole while others argue *pfcrt* binds directly to CQ, thereby inhibiting its ability to alter food vacuole pH.[60]

1.4.1.2. Non-clinical Safety of Chloroquine

Given the extensive clinical experience demonstrating the safety of CQ over the last 60 years, the available non-clinical information for chloroquine is limited and most studies are relatively old. No information was found in the literature concerning chloroquine administration to neonatal animals. The available non-clinical data for CQ suggest adverse events at doses 16-40 fold higher than those proposed for this malaria program.

1.4.1.2.1. Preclinical Safety of Chloroquine on Reproductive Systems

For CQ fertility/reproductive preclinical studies, limited information is available in the published literature. CQ can produce adverse embryonic effects/fetal abnormalities in experimental animal pregnancies, but at doses of CQ that are substantially higher than doses used for malaria prophylaxis or treatment.[61] Udalova et al[62] using a single dose of CQ 1000 mg/kg administered to pregnant rats on Day 9 of gestation reported an increase in embryonic deaths and fetal ocular malformations. Walker and Warner[63] reported that a single dose of CQ 400 mg/kg administered orally to rats between Days 8-9 of gestation produced mainly ocular malformations. Sharma and Rawat[64] orally administered CQ 700 mg/kg to rats on 15 days of gestation. A significant reduction in maternal body weight and placental weights were observed. Fetal growth retardation was also noted along with an increase in skeletal abnormalities. The authors concluded that the effects of CQ on the developing rat fetus are similar to that of ethanol. The above doses of CQ in animals are from 16- to 40-fold greater than the proposed dose of CQ 30 mg/kg body weight to be evaluated in the study.

090177e1811d3ccb\Approved\Approved On: 19-Feb-2010 20:59

090177e1811d3ccb\Approved\Approved On: 19-Feb-2010 20:59

1.4.2. Clinical Summary of Chloroquine

1.4.2.1. Clinical Pharmacology of Chloroquine

The pharmacokinetics of CQ was recently evaluated in healthy subjects in studies A0661118 and A0661186. Following oral administration, CQ is rapidly and almost completely absorbed from the gastrointestinal tract with mean peak plasma concentration ~5-7 hours. The mean plasma half-life of CQ is ~200 hours. CQ undergoes appreciable degradation in the body and the main primary active metabolite, desethyl-CQ, has a mean elimination half-life around 240 hours (10 days). Available CQ PK data suggests that there is no significant difference in the pharmacokinetics of CQ between pregnant and non-pregnant women and no dosage adjustment is required for CQ in pregnant women. Lee and colleagues[65] compared the pharmacokinetics of CQ and desethyl-CQ following administration of a standard 3 day 25 mg/kg dosing regimen of CQ in 12 pregnant and 13 non-pregnant patients with acute *P. vivax* malaria on the northwestern border of Thailand.[65] While the total area under the blood concentration-time curve ($AUC_{0-\infty}$) of CQ tended to decrease with the gestational age, the overall pharmacokinetics of CQ was not significantly different between pregnant and non-pregnant women. The AUC values for desethyl-CQ were not significantly affected by pregnancy either. The authors concluded that no dosage adjustment is required in pregnant women.[65]

1.4.2.2. Clinical Efficacy of Chloroquine Monotherapy in Malaria

Due to prevailing drug resistance in East Africa, CQ monotherapy is not used for chemoprophylaxis or treatment. It continues to offer some protective effect for pregnant women, however, in West Africa. A recent observational study in Benin examined the effect of self-administered CQ among pregnant women (N=1090), comparing self-reported dosing over pregnancy with birth weights. An estimated 49.9% of women reported taking weekly CQ in the first trimester, increasing to 92% of women in the second trimester and 97.5% in the final trimester. Subjects with self-reported chemoprophylactic use for seven or more months were four times more likely to give birth to child of normal birth weight (>2500 grams) than women who used chemoprophylaxis for less than four months (adjusted OR = 3.96; 95% CI = 1.9 to 8.28; p=<0.001).[66]

Although CQ is no longer recommended for treatment of *P. falciparum* due to high levels of resistance, particularly in East Africa, the return of parasite sensitivity to CQ has been documented in Malawi. In 1993, CQ in vivo failure rates in Malawi were as high as 58%.[67] Five years later, after changing to SP as first-line treatment, CQ inhibited in vitro blood schizont development in 96.5% (28 of 29) of isolates in Malawi,[68] indicating that *pfcrt* was no longer under selection pressure. In 2001, field sampling failed to find parasites carrying the *pfcrt* mutation associated with resistance[69] and a clinical trial using CQ monotherapy was 100% efficacious (63 of 63) among asymptomatic semi-immune adults who received 600 mg on Day 0 and Day 1, and 300 mg on Day 2.[70] Most recently, a study in 2005 showed CQ to clear parasite infection in 98.8% (79 of 80) of Malawi children with uncomplicated *P. falciparum* malaria.[71] The re-emergence of high *in vitro* sensitivity to CQ in Malawi, within just five years, suggests the *pfcrt* resistance mutation involves considerable fitness

cost to *P. falciparum*.[72-74] Kenya recently reported similar micro-evolutionary changes in P. *falciparum* since suspending CQ use in 1999, but at a slower rate than Malawi. Many reasons may contribute to delayed return of sensitivity, not the least of which is that CQ continued to be widely available for four years after the national treatment policy was changed, and the fact that amodiaquine, an analogue of CQ, was the second-line treatment during the CQ era and continues to be used to this day as the second-line therapy.[75]

Evidence of CQ efficacy was reported, as well, in a recent four-arm clinical trial conducted in Ghana among pregnant women with asexual *P falciparum* stage parasitemia. Women randomized to a CQ treatment group (N=225) received 600 mg for 2 days and 300 mg on the third day. The Day 28 treatment failure rate (PCR uncorrected) was 30% (62 of 208). Polymerase chain reaction (PCR) analysis confirmed that 14% (30 of 208) were true treatment failures (recrudescence) while 6% (11 of 208) were re-infections. PCR was unable to distinguish cases of recrudescence from new infection in the remaining 10% (21 of 208).[14] In a phase 2 treatment trial in India (066-191), CQ were given to patients with smears positive for *P. falciparum* at dose of CQ base 600 mg for first two days and 300 mg on the third day. The analysis indicated suboptimal efficacy (27% at Day 28) with CQ monotherapy.[53]

In addition, CQ may offer protection against maternal-to-child transmission (MTCT) of HIV. Cord blood containing high levels of CQ has been associated with a reduced risk of MTCT of HIV.[76] Viral shedding in breast milk has been lowered among HIV-positive women who received three days of 600 mg CQ as an anti-malarial chemoprophylactic.[77] It is unknown whether this reduction in viral load is sufficient to prevent HIV transmission among mothers who choose to breastfeed.

1.4.2.3. Clinical Safety and Tolerability of Chloroquine

CQ is safe and generally well tolerated in treatment doses. Due to its rapid absorption, CQ has a narrow therapeutic index, increasing the potential for toxic overdose. Hypotension and cardiac failure can be prompted by a single oral dose of 3500 mg,[78] six- fold higher than the IPTp proposed dose (600 mg). Despite toxicity at high doses, the most commonly reported side-effect in African populations is pruritus which is mostly mild in intensity and peaks 24 hours after intake.[79]

1.4.2.3.1. Clinical Safety of Chloroquine in Pregnancy

CQ has been widely used since the 1940s for the treatment of malaria and is included in the WHO and the US Centers for Disease Control and Prevention (CDC) guidelines for the treatment women in all trimesters of pregnancy infected with susceptible strains of the parasite. It has been demonstrated safe crossing the placenta without teratogenic effect.[80] A review of the medical literature regarding the use of CQ in pregnant women did not identify any adverse outcomes when pregnant women are treated in accordance with dosing regimens suggested in the WHO and the CDC guidelines for the treatment of malaria.

090177e1811d3ccb\Approved\Approved On: 19-Feb-2010 20:59

Three publications were identified that described clinical trials in pregnant women where CQ was administered, none reported any safety-related information in either women or their offspring to be of use to this review.[81-83]

Law and colleagues performed a study of cord/maternal drug distribution in 19 mothers who had been prescribed IPTp with CQ (750 mg daily) for 3 consecutive days and assessed breast-milk transfer following another course of CQ administered in the early postnatal period (Days 1–3 after delivery).[84] Absolute infant dose via milk (mg/kg/day) was calculated as the product of the milk Cavg and an average infant milk intake of 0.15 l/kg/day. Relative infant dose was calculated as absolute infant dose/days of exposure (mg/kg)/maternal dose (mg/kg) and expressed as a percentage. The median absolute and relative infant doses were 34 mg/kg/day and 2.3% for CQ and 15 mg/kg/day and 1.5% for desethyl-CQ, respectively. The absolute dose is well below recommended pediatric and neonatal treatment doses and the combined relative infant dose (CQ plus desethyl-CQ = 3.2%) is also significantly lower than the recommended 10% safety cut-off. The authors believe that this, in addition to the absence of observed adverse effects, is supportive of CQ's compatibility for use at recommended doses during breast-feeding.

1.5. Azithromycin-Chloroquine Combination (AZCQ)

1.5.1. Non-Clinical Summary Information

1.5.1.1. Non-Clinical Efficacy of the Azithromycin and Chloroquine Combination against *P. falciparum*

In an in vitro study by Ohrt et al,[85] AZ and CQ combination demonstrated additive to synergistic activity against eight CQ resistant P. *falciparum* isolates and exhibited an additive response against two CQ sensitive strains in vitro. Additive effects were also observed in studies conducted by Gingras[38] and Sidhu.[30] Fidock et al at Columbia University recently demonstrated potent in vitro synergy of AZ and CQ against CQ resistant *P. falciparum* strains from an investigational site in Bamako, Mali for Pfizer's adult treatment study A0661155 with the fractional inhibitory IC_{90} values of 0.3 to 0.4. (Personal communication, ASTMH, New Orleans, December 9, 2008).

One of the issues with the use of AZ in the treatment of malaria has been the slower rate of resolution of parasitemia, possibly a consequence of its mechanism of action (inhibition of protein synthesis). If combined with a fast-acting parasiticidal agent, the therapeutic utility of AZ may be optimized. The addition of a more rapidly cidal agent to AZ had been recommended as a way to optimize its application in a clinical setting. CQ has rapidly reduced the circulating burden of malaria parasites. A combination of CQ and AZ may therefore be rapidly parasiticidal while reducing the likelihood of breakthrough resistance.

1.5.1.2. Non-Clinical Safety of Azithromycin and Chloroquine Combination

Since CQ and macrolides (except AZ) are known to prolong the QT interval of electrocardiogram (ECG) measurements, the combination of AZ and CQ was evaluated in a non-clinical model to assess the arrythmogenicity potential of the combination. Alternans

090177e1811d3ccb\Approved\Approved On: 19-Feb-2010 20:59

(action potential duration alternations) is a measure of cardiac instability in humans and animals associated with the onset of ventricular fibrillation. Arrythmogenicity potential of CQ, AZ and combination of CQ and AZ were assessed in an anesthetized guinea pig alternans model following administration AZ and CQ alone and following combination treatment at clinically relevant concentrations proposed for the treatment/IPT of malaria. CQ alone, but not AZ, caused a profound increase in action potential duration. None of the drugs or their combination had any significant effects on alternans at the adjusted free drug concentrations studied. These concentrations represented 1- to 2-fold the Cmax levels with either drug alone or 10- to 15-fold the maximum free drug concentrations anticipated following dosing with CQ and AZ in clinical studies. Despite the ability of CQ to inhibit hERG current and prolong QT potential, it appears to lack proarrhythmic liability at concentrations at or exceeding clinically relevant levels alone or when used in combination with AZ.

1.5.2. Clinical Summary Information for Azithromycin and Chloroquine Combination

1.5.2.1. Clinical Pharmacology of Azithromycin and Chloroquine Combination

The pharmacokinetics of AZ and CQ was characterized in healthy subjects following co-administration of AZ and CQ tablets in healthy volunteers in study A0661118. AZ had no clinically relevant effect on CQ pharmacokinetics and CQ had no clinically relevant effect on AZ pharmacokinetics either. In a clinical pharmacology study A0661186, the systemic exposures (Cmax and AUClast) to AZ, CQ and desethyl CQ following fixed dose combination of AZCQ tablets were comparable to those achieved following administration of commercial standalone tablets of AZ (Zithromax) and CQ (Aralen) evaluated in earlier adult treatment studies, A0661134 and A0661155 (Table 2).

Table 2. PK Parameters from A0661186 Study Formulation

	**Cmax[a] (ng/ml)**	**AUClast[a] (ng.hr/ml)**
**Chloroquine**		
Azithromycin 500 mg and Chloroquine base 300 mg individual tablets	108 (34)	3281 (30)
Azithromycin and Chloroquine base 250/150 mg X 2 tablets	95.8 (36)	3253 (35)
**Azithromycin**		
Azithromycin 500 mg and Chloroquine base 300 mg individual tablets	437 (38)	3877 (32)
Azithromycin and Chloroquine base 250/150 mg X 2 tablets	496 (40)	3910 (30)

[a] Geometric mean (CV%)

090177e1811d3ccb\Approved\Approved On: 19-Feb-2010 20:59

In a recently published study, Salman and colleagues evaluated the pharmacokinetic properties of AZ for its use in combination with CQ for IPTp in 31 pregnant and 29 age-matched non-pregnant Papua New Guinean paticipants. All study participants received two 2000 mg doses of AZ that were given 24 hours apart to pregnant and age-matched non-pregnant participants in combination with 450 mg CQ base daily for three days. The results demonstrated that in pregnant and non-pregnant participants, AZ exposures ($AUC_{0-\infty}$:28.7 vs 31.8 mg·h/L respectively) and the estimated terminal elimination half-lives (78 vs 77 hours, respectively) were similar.[86] The characterized pharmacokinetics of AZ in this study are also consistent with the results of pervious studies.[86] This study did not evaluate CQ pharmacokinetics.

1.5.2.2. Clinical Efficacy of Azithromycin and Chloroquine Combination against *P. falciparum*

The clinical efficacy of AZ and CQ co-administration was investigated in several Phase 2/3 clinical studies. The efficacy outcomes from these studies are summarized below.

- A two-stage Phase 2 treatment trial in India demonstrated in vivo synergy between AZ and CQ against P. *falciparum* infection.[53] In the first stage of the trial (066-191), neither CQ monotherapy (27% efficacy at Day 28) nor AZ monotherapy (33% efficacy at Day 28) demonstrated adequate efficacy. During the second stage (066-191B), the combination of AZ and CQ demonstrated a 97% efficacy at Day 28. The dosing regimen used in this study was 1000 mg AZ once daily and 600 mg CQ base administered once daily for 3 days.

- Subsequently, the combination of AZ and CQ was evaluated for acute uncomplicated *falciparum* malaria in three Phase 2/3 studies, A0661120, A0661126 and A0661154 in India and South America regions. A dose response for AZ (on mg/kg basis) was observed. While efficacy rates observed in India (A0661120) and South America (A0661126) were suboptimal (86.3% and 57.1% respectively) with 1000 mg of AZ dose administered once daily for 3 days, the efficacy rate with the regimen containgin higher AZ dose of 2000 mg in a subsequent study in A0661154 was 97.2% at Day 28 (96.4% at Colombia and 98.1% at India).[87]

- Two further phase 2b/3 randomized comparative studies (A0661134 and A0661155) were conducted in sub-Saharan Africa in adults with the combination regimens of 1000 mg AZ and 600 mg CQ, once daily for 3-day treatment. Study 1134 was conducted in five countries in Africa including Ghana, Mali, Zambia, Kenya and Uganda. Study 1155 was conducted in Ghana, Mali, Zambia, Burkina Faso and Senegal. Both studies demonstrated efficacy, safety, and tolerability in the treatment of acute uncomplicated *falciparum* malaria. The efficacy rate was 98% (Appendix 1) and 100% on Day 28 for study A0661155 respectively. Both studies demonstrated efficacy, safety, and tolerability in the treatment of acute uncomplicated *falciparum* malaria. The Day 28 efficacy rates were 98%[88] and 100%[89] (PCR corrected), and 95% and 99% (PCR uncorrected) for study A0661134 and A0661155, respectively.

090177e1811d3ccb\Approved\Approved On: 19-Feb-2010 20:59

- A pediatric Phase 2/3 clinical trial (A0661157) of AZCQ is ongoing in Kenya, Mali, Ghana, Burkina Faos and Cote d'voire. This is a comparative study designed to evaluate a fixed dose AZCQ combination regimen of 30 mg/kg of AZ plus 10 mg/kg of CQ base once daily for 3 days vs. standard regimen of AL (artemether/lumefantrine) in treatment of symptomatic uncomplicated malaria. The study is being conducted in two sequential age-based cohorts. Enrollment in the first cohort older subjects between ≥5 years of age and ≤12 years of age is complete. Enrollment in the primary target Cohort 2, with younger subjects between ≥6 months of age to ≤59 months of age is ongoing. A blinded interim analysis (IA) was recently conducted at 50% completion of Cohort 2. The Based on review of IA data, the Independent Data Monitoring Committee (IDMC) for this program recommended to continue with the study.

1.5.2.3. Clinical Safety and Tolerability of Azithromycin and Chloroquine Combination

AZ and CQ are marketed compounds with well established safety profiles in adults, children and pregnant women. The co-administration of AZ and CQ at doses up to 2000 mg (~30 mg/kg) AZ plus CQ 600 mg base (~10 mg/kg) daily for three days appears well tolerated based on the data from 066-191/b, A0661120, A0661118, A0661126, A0661134, A0661139, A0661154 and A0661155 studies. Phase 1 trial A0661139 demonstrated comparable tolerability between the combination of 1000 mg and CQ 600 mg (base) daily for three days and the combination of 1500 mg and CQ 600 mg base daily for three days. Most adverse events reported with AZ and CQ co-administration were mild in nature and were related to GI tract. Pruritus is an adverse event commonly associated with CQ use in African populations, and was frequently observed in A0661134 and A0661155 studies conducted in Africa.

CQ is known to delay cardiac repolarization through inhibition of the rapidly activating delayed rectifier potassium current known as hERG and treatment with CQ has been shown to cause QT prolongation in patients. Macrolides (other than AZ) are also known to prolong QT interval on ECG measurements, however, this effect has not been observed with AZ. In the cardiovascular safety study A0661139, co-administration with AZ did not significantly affect the QTc effect associated with CQ use. Use of AZ or CQ has not been associated with torsades de pointes, a type of fatal arrhythmia often associated with drugs that cause QT prolongation. Refer to the Investigator's Brochure (IB) for details.

1.5.2.3.1. Clinical Safety of Azithromycin and Chloroquine Combination in Pregnancy

Both AZ and CQ have been widely used in all trimesters of pregnancy and are considered safe in pregnant women as individual agents. AZ has been used in all trimesters of pregnancy for STI and bacterial infections with dosage between 500 mg and 2,000 mg.[90] CQ was recommended for prevention and treatment of malaria in all trimesters of pregnancy until recently with CQ resistance becoming widespread and the treatment courses with less than 2000 mg CQ base (total dose) produced similar rates of adverse events.[91]

090177e1811d3ccb\Approved\Approved On: 19-Feb-2010 20:59

A comprehensive review of the literature to identify publications describing the safety of CQ and AZ in pregnant women did not identify any additional safety issues beyond the information contained in the CQ and AZ product labels. In the doses used for malaria treatment and prophylaxis in pregnant women, an increased risk of adverse pregnancy outcomes has not been reported with CQ. With regard to AZ, many years of worldwide use, in addition to randomized trials and observational epidemiology studies have not identified any adverse pregnancy outcomes associated with AZ exposure during pregnancy.

To date there have been no well-controlled clinical studies evaluating the effects of co-administration of AZ and CQ in pregnant women also containing safety information. In Pfizer trials where both drugs were used concomitantly for the treatment of uncomplicated malaria in adults, no additional safety concerns beyond those listed in the labels of the individual products were identified. No pharmacokinetic interaction is observed between the two drugs.

Please refer to the IB for additional information regarding the use of AZ and CQ combination in Malaria.

1.6. Rationale

A fixed dose combination of AZ and CQ (AZCQ) is being developed as a potential replacement of SP for IPTp in sub-Saharan Africa. To evaluate the superiority of the AZCQ IPTp regimen over the SP IPTp regimen, a larger study, A0661158, is planned in countries in East and Southern sub-Saharan Africa, where SP is the current standard of care and SP resistance is a considerable issue. This will be the pivotal study for regulatory submissions to the EMA and to the national regulatory agencies in Sub-saharan African countries.

AZCQ is expected to exert its IPTp effect through several mechanisms. They include peripheral parasitological clearance, prevention of re-infections, prevention/treatment of placental parasitemia and prevention/treatment of STIs. The relative contribution of each of these mechanisms is not known. This study, A0661201, is a non-comparative parasitological clearance study of a single 3-day treatment course of AZCQ in about 166 pregnant women with asymptomatic parasitemia. The results from his study will help to characterize the magnitude of parasitological clearance required for the IPTp effect observed in pivotal A0661158 study and will be used as a supportive study for the regulatory submissions.

From PK studies mentioned earlier in PK sections, AZ and CQ have not demonstrated any significant drug interaction in non-pregnant healthy volunteers. PK evaluations of AZ and CQ do not suggest any need for dose adjustment in pregnant women. The exposures (AUClast) of AZCQ fixed dose were comparable to those of the individual tablets of AZ and CQ in non-pregnant healthy subjects. However, since the pharmacokinetics of the combination tablet has not been evaluated in pregnant subjects, study A0661201 will also evaluate PK exposures of AZCQ combination in pregnant subjects.

090177e1811d3ccb\Approved\Approved On: 19-Feb-2010 20:59

1.6.1. Dosing Regimen Selection (AZCQ)

The study drug AZCQ is a fixed dose combination tablet of AZ and CQ containing 250 mg AZ and 155 mg CQ base. The dosing regimen will consist of four AZCQ tablets (a total of 1000 mg AZ/620 mg CQ base), *per os* (PO, orally) once daily for three days (Days 0, 1, 2).

The above regimen has been chosen since this constitutes one treatment course of the AZCQ IPTp regimen that is planned to be evaluated in the pivotal IPTp study A0661158. The full IPTp regimen for AZCQ consists of three such treatment courses mentioned above administered during second and third trimesters of pregnancy with 4-8 weeks between two consecutive treatment courses.

Rationale for AZCQ Dosing Regimen: The dosing regimen of AZCQ has been selected for the IPTp program for the following reasons:

1. The above 3-day dosing regimen (four AZCQ tablets (a total of 1000 mg AZ/620 mg CQ base), *per os* (PO, orally) once daily for three days (Days 0, 1, 2) was evaluated and proved efficacious in two adult treatment clinical trials (A0661134 and A0661155) in sub-Saharan Africa. Both PCR corrected and uncorrected parasitological efficacy rates were above 90%. For details on studies A0661134 and A0661155, please refer to Section 1.5.2.2. and the IB.
2. In a clinical pharmacology study in healthy subjects (A0661186), the pharmacokinetics of the fixed dose AZCQ combination tablet appeared to be comparable to that of the individual tablets of AZ and CQ used in earlier adult treatment trials.
3. Although pregnant women experience unique physiological changes that can alter drug disposition during gestation, available data suggests that AZ and CQ PK are not significantly different between pregnant and non-pregnant women.
4. A total AZ dose of 3000 mg to be used for each IPTp treatment course is higher than the 2000 mg dose needed for treatment of STIs including syphilis and *N. gonorrohoeae*.

A fixed dose combination tablet formulation of AZ and CQ, AZCQ (250 mg AZ/155 mg CQ base) has been developed specifically for the IPTp indication. Each tablet is labeled to contain 250 mg AZ and 155 mg CQ base. Please note that the content of CQ base in this tablet is equivalent to that contained in the commercial tablets of CQ (Aralen, 150 mg CQ base) used in the previous studies (A0661134 &A0661155). CQ is present in these tablets in the form of its phosphate salt. The 5 mg difference in strength is explained by the use of slightly different (more accurate) CQ base potency factors for in the CQ phosphate salt than what is used for the Aralen tablets. The combination tablet uses the actual potency factor (62.0%) vs Aralen that uses an approximate number of 60% for potency factor.

090177e1811d3ccb\Approved\Approved On: 19-Feb-2010 20:59

2. STUDY OBJECTIVES AND ENDPOINTS

2.1. Objectives

The primary objective is to evaluate the peripheral parasitological clearance rate of AZCQ on Day 28 (PCR corrected) following first dose of 3-day dosing regimen of AZCQ in asymptomatic pregnant women with *P. falciparum* parasitemia.

Secondary objectives include the evaluation of:

1. Parasitological clearance rates (PCR corrected) at Days 7, 14, 21, 35, and 42 post first dose of study medication;

2. Parasitological clearance rates (PCR uncorrected) at Days 7, 14, 21, 28, 35, and 42 post first dose of study medication;

3. Pharmacokinetic exposure of AZCQ;

4. Safety and tolerability of AZCQ;

2.2. Endpoints

Primary Efficacy Endpoint

- Parasitological response (PCR corrected) at Day 28 post first dose of study medication.

Secondary Efficacy Endpoints

- Parasitological responses (PCR corrected) at Days 7, 14, 21, 35, and 42 post first dose of study medication;

- Parasitological responses (PCR uncorrected) at Days 7, 14, 21, 28, 35, and 42 post first dose of study medication;

- Parasite counts at each visit– number of asexual *P. falciparum* parasites per microliter of blood.

Pharmacokinetic Evaluation

- Pharmacokinetic exposure of AZCQ.

Safety Endpoints

- Safety and tolerability endpoints including spontaneously reported adverse events, temperature, hemoglobin concentrations, and the EIU assessment.

090177e1811d3ccb\Approved\Approved On: 19-Feb-2010 20:59

090177e1811d3ccb\Approved\Approved On: 19-Feb-2010 20:59

3. STUDY DESIGN

This is an open label, single arm non-comparative out-patient study in pregnant women during their second and third trimesters of pregnancy. Women will be screened for peripheral parasitaemia. Women who are screened but not included in the study (screen failures) will be referred to their doctors and will be given standard antenatal care (ANC) including IPTp. Subjects with asymptomatic parasitemia (counts of 80 -100,000/ µL) will receive a single 3-day course of AZCQ IPTp regimen. Subjects will be followed up on a weekly basis up to Day 42 after the first dose, and following delivery or at termination of pregnancy for EIU safety assessments. After completing Day 42 evaluation, all subjects will continue to receive standard antenatal care including IPTp with SP if the gestational age allows additional IPTp course(s). The parasitological response will be evaluated on Days 7, 14, 21, 28, 35 and 42. Pharmacokinetics evaluation will be conducted on blood samples collected from the subjects who consented for such test. Systemic concentrations of AZ, CQ and desethyl-CQ will be evaluated on Day 0 predose, Day 2 predose, 2 hours (as close to 2 hours as possible) and 8 hours (window: 4 to 12 hours) post dose, and at a random time point on Days 7 and 14. In addition, due to the long half-life of CQ, systemic concentrations of CQ and desethyl-CQ will also be measured at a random time point on Days 21 and 28. All subjects will also be followed up for EIU safety assessments following delivery or termination of pregnancy. The insecticide treated bed-nets will be given to all subjects on Day 0 of the study with installation verified during Day 1 home visit by fieldworker(s).

4. SUBJECT SELECTION

This study can fulfill its objectives only if appropriate subjects are enrolled. The following eligibility criteria are designed to select subjects for whom protocol treatment is considered appropriate. All relevant medical and non-medical conditions should be taken into consideration when deciding whether this protocol is suitable for a particular subject. Subjects who fail to meet the selection criteria for the study will be referred to appropriate physician for receiving standard ANC care including IPTp treatment as per national/local antenatal care guidelines.

4.1. Inclusion Criteria

Subject eligibility should be reviewed and documented by an appropriately qualified member of the investigator's study team before that subject is included in the study.

Subjects must meet all of the following inclusion criteria on Day 0 to be eligible for enrollment into the study:

1. Primigravidae and secundigravidae pregnant women at ≥14 and ≤30 weeks of gestational age (confirmed by ultrasound examination).
2. Evidence of asymptomatic parasitemia with Plasmodium falciparum monoinfection (confirmed by microscopy) with parasite counts in the range of 80-100,000/µL on thick blood smears.

3. Evidence of a personally signed and dated informed consent document indicating that the subject (or a legally acceptable representative if a subject is <18 years of age) has been informed of all pertinent aspects of the study and that all questions by the subject have been sufficiently answered. Assent will be obtained from subjects <18 years of age.

4. Subjects who are willing and able to comply with scheduled visits, treatment plan, laboratory tests, and other study procedures.

5. Subjects who agree to be supervised for treatment administration, and are available for all follow up visits as per protocol.

4.2. Exclusion Criteria

Subjects presenting with any of the following will not be randomized in the study:

1. Age <16 years old or >35 years old.

2. Multiple gestations (more than one fetus) as per the ultrasound results at screening.

3. Clinical symptoms of malaria.

4. Hemoglobin <8 g/dL (measured at baseline).

5. Any condition requiring hospitalization or evidence of severe concomitant infection at time of presentation.

6. Use of antimalarial drugs in previous 4 weeks.

7. History of convulsions, hypertension, diabetes or any other chronic illness that may adversely affect fetal growth and viability.

8. Inability to tolerate oral treatment in tablet form.

9. Known allergy to the study drugs (AZ, CQ, and SP) or to any macrolides or sulphonamides.

10. Present history of smoking or alcohol or drug abuse since becoming pregnant.

11. Participation in other studies within 30 days before the current study begins and/or during study participation.

12. Inability to comprehend and/or unwillingness to follow the study protocol.

13. Concurrent participation in another investigational study.

14. Previously randomized in this study.

090177e1811d3ccb\Approved\Approved On: 19-Feb-2010 20:59

15. Requirement to use medication during the study that might interfere with the evaluation of the study drug of AZ or CQ or is contra-indicated during pregnancy per package inserts.

16. Severe acute or chronic medical or psychiatric condition or laboratory abnormality that may increase the risk associated with study participation or investigational product administration or may interfere with the interpretation of study results and, in the judgment of the investigator, would make the subject inappropriate for entry into this study. Examples would include but not limited to:

    a. Symptomatic HIV infection, including physical findings that suggest immunocompromised status (eg, oral candidiasis);

    b. Neurological conditions which may predispose to complications during pregnancy, including seizure disorders;

    c. Severe psychosis or major disorder that may interfere with the conduct of the study or adherence to study medication;

    d. Known, clinically significant pre-existing renal or hepatic disease.

17. Evidence of current obstetric complications that may adversely impact the pregnancy and/or fetal outcomes, including presence of congenital anomalies, placenta previa or abruption.

18. Known severe sickle cell (SS) disease or sickle-hemoglobin C (SC) anemia.

19. Known family history of prolonged QT syndrome, serious ventricular arrhythmia, or sudden cardiac death.

4.3. Randomization Criteria

Since this is a non-comparative single arm study, there is no randomization. The subjects who meet all inclusion/exclusion criteria will receive study medication at the Day 0 visit. Subjects will be assigned patient identification numbers (PID numbers) sequentially at the Day 0 visit. This unique PID number will be retained throughout the study and must appear on all CRF pages, source documents and lab data.

Study drug will be administered as open label treatment under supervision.

4.4. Life Style Guidelines

Subjects will be encouraged to stay within the area during the study period and deliver in a hospital setting. Subjects will be encouraged to use LLINs. Subjects will be followed up for EIU safety assessments following delivery or termination of pregnancy. After completing Day 42 evaluation, all subjects will continue to receive standard antenatal care including IPTp with SP if the gestational age allows additional IPTp course(s).

090177e1811d3ccb\Approved\Approved On: 19-Feb-2010 20:59

5. STUDY TREATMENTS

Study drug AZCQ is a fixed dose combination tablet of AZ and CQ containing 250 mg AZ and 155 mg CQ base. The dosing regimen to be evaluated in this study will consist of four AZCQ tablets (a total of 1000 mg AZ/620 mg CQ base), given orally once daily for 3 days (Days 0, 1, 2). The first dose on Day 0 and the third dose on Day 2 will be administered under supervision at the antenatal care facility (ANC), and the second dose on Day 1 will be administered at home by under supervision of a field worker.

Contraindications

The site investigator and study staff must be thoroughly aware of all contraindications and other information contained in the Package Inserts for AZ (Zithromax) and CQ (Arelen), and IB for the study medication AZCQ.

5.1. Allocation to Treatment

The investigator's knowledge of the treatment should not influence the decision to enroll a particular subject. Subjects who meet all the inclusion/exclusion criteria will be allocated to the treatment of AZCQ on Day 0.

5.2. Breaking the Blind

The study is open label.

5.3. Drug Supplies

All trial medication (AZCQ) will be supplied by Pfizer. AZCQ will be supplied as a combination tablet containing 250 mg AZ and 155 mg CQ base.

5.3.1. Formulation and Packaging

AZCQ fixed-dose combination tablets will be supplied in unit dose bottles which contain 4 tablets each and are non-patient specific.

5.3.2. Preparation and Dispensing

Only qualified personnel who are familiar with procedures that minimize undue exposure to them and to the environment should undertake the preparation, handling, and safe disposal of chemotherapeutic agents.

AZCQ will be administrated to a subject under supervision by the investigator or the fieldworker once daily for three days of therapy.

090177e1811d3ccb\Approved\Approved On: 19-Feb-2010 20:59

5.3.3. Administration

Study medication will be administered as open label treatment under supervision. Study medication will be dispensed by the investigator or the fieldworker (Section 5.3.2). AZCQ should not be administered on an empty stomach. Each dose should be administered with a glass of water. First and third doses will be administered under supervision by investigator during ANC visits. The second dose will be administered at home under the supervision of fieldworker(s).

Any dose that is vomited within 30 minutes of administration will be repeated. If vomiting re-occurs after re-dosing on Day 0, Day 1 and Day 2, the subject will not receive further study treatments and will be provided standard treatment for malaria in pregnancy. The standard ANC care including IPTp will be given to the subject per local ANC guideline. If this happens during the home visit on Day 1, subject will be asked to return to the study physician within next 24 hours and will receive standard treatment for malaria in pregnancy. The subject will be followed through Day 42 and for pregnancy outcomes after delivery or termination of pregnancy for EIU safety assessments.

5.3.4. Compliance

The medication will be administered to the subject under supervision by investigators during ANC visit or at home under supervision by the fieldworkers.

5.4. Drug Storage and Drug Accountability

The investigator, or an approved representative (eg, pharmacist), will ensure that all study drug is stored in a secured area, under recommended storage conditions and in accordance with applicable regulatory requirements. Prior to dispensing, all study medications should be stored under the conditions per label instructions. The investigator or the fieldworker will be responsible for recording the receipt and usage of all drugs supplied and for ensuring the supervision (via the hospital pharmacist or fieldworkers where relevant) of the storage and allocation of these supplies. To ensure adequate records, all study drugs will be accounted for in the case report form and drug accountability inventory forms as instructed by Pfizer.

The investigator or the fieldworker must maintain adequate records documenting the receipt, use, loss, or other disposition of the investigational products. Pfizer will supply drug accountability forms that must be used. The forms must identify the investigational products, including batch or code numbers, and account for its disposition on a subject-by-subject basis, including specific dates and quantities. The forms must be signed by the individual who dispensed the drug, and copies must be provided to Pfizer.

At the end of the trial, Pfizer will provide instructions as to disposition of any unused investigational products. If Pfizer authorizes destruction at the trial site, the investigator must ensure that the materials are destroyed in compliance with applicable environmental regulations, institutional policy, and any special instructions provided by Pfizer. Destruction must be adequately documented.

090177e1811d3ccb\Approved\Approved On: 19-Feb-2010 20:59

5.5. Concomitant Medication(s)

The medications, not contra-indicated during pregnancy and not contra-indicated for co-administration with AZ or CQ, may be used through Day 42 following the first dose of AZCQ. After Day 42, the medications not contra-indicated during pregnancy may be used. All medications used by the subject at study entry or at any time through last study visit following the delivery or the termination of pregnancy must be recorded in the case report form. At each study visit, all medications taken since the last evaluation will be recorded.

While interactions with digoxin, ergot alkaloids, triazolam, carbamazepine, cyclosporine, hexobarbital and phenytoin have not been reported in clinical trials with AZ, they have been observed with macrolide products. Careful monitoring of subjects taking these medications is advised.

A single oral dose of AZ co-administered with nelfinavir at steady-state resulted in increased AZ serum concentrations. Although it is not necessary to adjust the dose of AZ when administered in combination with nelfinavir, close monitoring for known side effects of AZ (eg, liver enzyme abnormalities, hearing impairment) is warranted.

There have been reports which may indicate an increase in incidence and severity of adverse reactions when CQ is used with Fansidar as compared to the use of Fansidar alone (refer to Fansidar package insert).

Please refer to package inserts for AZ and CQ, and IB for AZCQ for further information regarding potential interactions between study drugs and other treatment agents

6. STUDY PROCEDURES

6.1. Study Period

6.1.1. Baseline/Day 0 Visit (at ANC clinic/center)

<u>Baseline/Day 0 – Pre Assessment of Study Eligibility</u>

After informed consent is obtained, the subject must have the following procedures performed prior to the start of therapy:

- Medical history;
- Obstetrical history;
- Urine pregnancy test (if not done at any prior ANC visit for this pregnancy);
- Concomitant treatment history;
- Rapid diagnostic test for *P. falciparum* (eg, Binax, See Appendix 1);

090177e1811d3ccb\Approved\Approved On: 19-Feb-2010 20:59

- Complete physical examination;
- Oral Temperature;
- Routine obstetric checkup;
- Obstetrical ultrasound (if urine pregnancy test positive);
- Thick and thin peripheral blood smears for parasite identification and counts (2 smears per subject) in subjects with Rapid Diagnostic Test positive for *P. falciparum*;
- Hemoglobin level (with HemoCue^{TM});
- Urine analysis for glucose and proteins;
- A dried blood blot for molecular genotyping (reinfection vs. recrudescence, eg, MSP1/MSP2) of parasite and for genetic markers of CQ resistance (eg, PfCRT);
- Two predose blood samples (one for AZ and one for CQ and desethyl-CQ analyses) from the subjects who consented for PK analysis (See Section 7.3 Pharmacokinetic Assessments). The exact date and time of blood sample collection will be recorded.

<u>Day 0 – Post Assessment of Eligibility</u>

Subjects who have met all inclusion/exclusion requirements will have the following procedures performed:

- Administer treatment (AZCQ);
- Assess adverse events;
- Distribute LLINs.

6.1.2. Days 1 Home Visit (by the field worker)

- Assess adverse events;
- Concomitant treatment history;
- Oral temperature: will be taken by the fieldworker;
- If a subject presents with symptoms of malaria (fever >37.5°C, oral), no further AZCQ dose would be given. The subject would be immediately referred to the investigator for parasite counts and appropriate treatment for malaria in pregnancy as per national/ local guidelines;

090177e1811d3ccb\Approved\Approved On: 19-Feb-2010 20:59

- Administer treatment: second dose of AZCQ will be administered at home under supervision by the fieldworker(s);
- Confirm that the LLINs has been installed in the home of the subject.

6.1.3. Days 2 Visit (at ANC clinic/center)

- Assess adverse events;
- Concomitant treatment history;
- Oral temperature;
- If a subject presents with symptoms of malaria (fever >37.5°C, oral), no further AZCQ dose would be given. The subject would be immediately referred to the investigator for parasite counts and appropriate treatment for malaria in pregnancy as per national/ local guidelines;
- Administer treatment: third dose of AZCQ;
- Two blood samples (one for AZ and one for CQ and desethyl-CQ analyses) from the subjects who consented for PK analysis at each of the following timepoints: predose, 2 hours (as close to 2 hours as possible) and 8 hours (window: 4 to 12 hours). The exact date and time of blood sample collection will be recorded.

6.1.4. Follow-up Visits on Days 7, 14, 21, 28, 35, 42 at ANC clinic/center)

- Assess adverse events;
- Concomitant treatment history;
- Limited physical examination (only on Days 42);
- Oral temperature;
- Hemoglobin (with HemoCue™) (only on Day 42);
- Thick and thin peripheral blood smears for parasite identification and counts (2 smears at each visit);
- If a subject presents with symptoms of malaria (fever >37.5°C, oral), and has parasitemia (microscopy), she will receive standard antimalarial treatment according to the local treatment guidelines for malaria in pregnancy;
- A dried blood blot will be collected at each visit for potential molecular testings;

090177e1811d3ccb\Approved\Approved On: 19-Feb-2010 20:59

- Two blood samples (one for AZ and one for CQ and desethyl-CQ analyses) on Days 7 and 14 (a random time point) from the subjects who consented for PK analysis. The exact date and time of blood sample collection will be recorded;
- One blood sample (for CQ and desethyl-CQ analyses only) on Days 21 and 28 (a random time point) from subjects who consented for PK analysis. The exact date and time of blood sample collection will be recorded.

6.1.5. Unscheduled Visits through Day 42

Any subject who develops signs and symptoms of malaria (fever (>37.5°C, oral) and new signs and symptoms of malaria including malaise, headache or abdominal pain at any time other than the scheduled visits through Day 42, will be required to report to the investigator/study staff immediately for further evaluation. Blood smears and blood blots will be collected. The subject will be treated with the current standard of care for malaria in pregnancy.

In the event that subjects require an unscheduled visit (eg, for additional follow up due to any safety concerns, concomitant illness etc), the following assessments/procedures should be completed at a minimum:

- Symptom-directed physical exam with temperature;
- Adverse event assessment;
- Concomitant treatment history.

6.1.6. Safety Follow-up Visit for EIU Assessment

Every attempt should be made to follow up the subject following delivery or at termination of pregnancy for EIU safety assessment. In case of no associated serious adverse events (SAE), subjects should be followed up by fieldworkers at clinic or at home within 14 calendar days after expected date of delivery or termination of pregnancy.

A. Following data will be collected at this visit:

- Concomitant treatment history;
- Maternal information and delivery: any history of complications during pregnancy, at and after delivery, eg, pre-eclampsia, hemorrhage, infection, etc;
- Neonatal Information (See Section 7.2 for details):
  - Outcome of pregnancy;
  - Outcome of infant.

090177e1811d3ccb\Approved\Approved On: 19-Feb-2010 20:59

When feasible and relevant for the assessment of the case (eg, in case of neonatal malformation/anomalies, premature delivery, low birth weight neonates, developmental assessment, kernicterus, or any other neonatal illness, hospitalization, drug therapy etc), further follow-up of neonates should continue until the information is satisfactory or the outcome has been established or the condition has stabilized.

B. In the event of an elective termination, spontaneous abortion, and late fetal death, following details should be recorded if available:

- Reason for termination;
- Gestational age at termination;
- Results of physical examination (gender, external anomalies) and pathology.

Any additional tests, procedures, treatments, and consultations may be conducted if recommended by local/national ANC guidelines and deemed clinically indicated by the investigator.

Any serious adverse events (SAEs) occurred after the informed consent through one month after the delivery or the termination of pregnancy should be reported to the study doctors immediately. Refer to Section 8.2 for details.

6.2. Subject Withdrawal

Subjects may withdraw from the study at any time at their own request, or they may be withdrawn at any time at the discretion of the investigator or sponsor for safety, behavioral, or administrative reasons. If a subject does not return for a scheduled visit, every effort should be made to contact the subject. In any circumstance, every effort should be made to document subject outcome and pregnancy outcome, if possible. The investigator should inquire about the reason for withdrawal, request the subject to return all unused investigational product(s), request the subject to return for a final visit, if applicable, and follow-up with the subject regarding any unresolved adverse events.

If the subject withdraws from the study and also withdraws consent for disclosure of future information, no further evaluations should be performed and no additional data should be collected. The sponsor may retain and continue to use any data collected before such withdrawal of consent.

Unless a subject withdraws the consent or leaves the town/village, she should continue in the study for follow up visits through Day 42, and following delivery or termination of pregnancy for EIU safety assessment. Subject may be withdrawn from the study treatment (during the first three days) due to safety concerns (eg, adverse events, significant laboratory abnormalities, and contraindications of concomitant medications), but not from the clinical study per se unless the subject withdraws consent. The standard antenatal care including IPTp will be given to the subject per local ANC guideline. The circumstances and data surrounding these events should be clearly documented in the case report form. The

090177e1811d3ccb\Approved\Approved On: 19-Feb-2010 20:59

investigator must determine the primary reason for discontinuation. A discontinuation must be reported immediately to the Pfizer medical monitor or his/her designated representative if it is due to a serious adverse event (SAE).

7. ASSESSMENTS

All study related assessment will be performed after obtaining informed consent document, and assent as appropriate.

7.1. Parasite Clearance Assessments

The primary tool for assessing the parasitological clearance is the peripheral blood smear (thick and thin), which will be prepared at the study sites with the standard Giemsa staining for parasite identification and count using white blood cell counting method on thick smears. Each blood smear will be read by two microscopists in a blinded fashion (blinded to the reading of other microscopists). In the event of discrepancy, the slide will be read by third microscopist and parasite density will be calculated by averaging the two most concordant counts. All the smears will be preserved for possible third party confirmation. Refer to the Laboratory Manual for details. If and when the blood smears become positive after initial parasite clearance, the blood blot collected at that visit will be tested for molecular genotyping assays to differentiate the recrudescence from reinfection.

- Recrudescence: Reappearance of asexual P. falciparum blood stage parasites confirmed by the results of molecular testing. This finding reflects that the subject failed the treatment since the original parasite recurred;
- Reinfection: Infection by a different genotypic parasite as documented by molecular testing. This finding reflects that the subject did not fail treatment since a molecularly distinct parasite was found at the time of recurrence.

7.2. Safety Assessments

Safety and tolerability will be assessed by spontaneously reported adverse events, history of concomitant treatments, temperature, hemoglobin concentrations, and EIU safety assessment according to Schedule of Activities.

- Spontaneous adverse events reporting: through Day 42. Refer to Section 8 (Adverse Event Reporting) for details;
- History of concomitant treatment assessment;
- Oral temperature through Day 42;
- Hemoglobin levels: with HemoCueTM, via finger stick or peripheral blood collection) at baseline and on Day 42.
- EIU Assessments:

090177e1811d3ccb\Approved\Approved On: 19-Feb-2010 20:59

Every attempt should be made to follow up the subject after the delivery for EIU assessment. In case of no associated serious adverse events (SAE), subjects should be followed up by fieldworkers at clinic or at home within 14 calendar days after expected date of delivery or termination of pregnancy.

- Maternal information and delivery: any history of complications during pregnancy, at and after delivery, eg, pre-eclampsia, hemorrhage, infection, etc.
- Neonatal information:
  - Outcome of pregnancy:
    - Full term live birth;
    - Premature live birth;
    - Still birth;
    - Spontaneous abortion/miscarriage;
    - Induced or elective abortion;
    - Gestational age at birth.
  - Outcome of infant:
    - Normal neonate;
    - Congenital malformation/anomaly;
    - Other neonatal problem/abnormality: include dysmaturity, neonatal illness, hospitalization, drug therapies.

  Infant details: include sex, weight at birth, length at birth, head circumference at birth, APGAR scores (1/5 minutes).

  - Fetal information will be collected in the event of pregnancy termination.
  - Additional information and further follow-up on the neonate will be conducted when feasible and relevant to the assessment of the case if applicable.

7.3. Pharmacokinetic Assessments

Blood samples will be collected from the subjects who consented for PK assessments. Followings are the sampling time points:

090177e1811d3ccb\Approved\Approved On: 19-Feb-2010 20:59

- Day 0 prior to dosing of AZCQ (window: -1 to 0 hour); collect blood samples for determination of AZ, CQ, and desethyl-CQ concentration;

- Day 2: prior to dosing of AZCQ (window: -1 to 0 hour), 2 hours (as close to 2 hours as possible) and 8 hours (window: 4 to 12 hours) post dose, as feasible; collect blood samples for determination of AZ, CQ, and desethyl-CQ concentration;

- Days 7, 14, 21 and 28: a random time point during the visits; collect blood samples for determination of AZ, CQ, and desethyl-CQ on Days 7 and 14; collect blood samples only for determination of CQ and desethyl-CQ concentration on Days 21 and 28.

Actual sampling date and time should be recorded for all samples. Subjects who received even one dose will be followed up for all subsequent PK sample collection.

7.3.1. Serum for Analysis of Azithromycin

Blood samples (3 mL) to provide a minimum of 1 mL serum for pharmacokinetic analysis of AZ will be collected into appropriately labeled tubes containing no preservative or anticoagulant or serum separator at protocol-specified times (See Section 6.1). All efforts will be made to obtain the PK samples at the exact nominal time relative to dosing. However, samples obtained within 10% of the nominal time (eg, within 6 minutes of a 60 minute sample) from dosing will not be captured as a protocol deviation, as long as the exact time of the sample collection is noted on the source document and data collection tool (eg, CRF).

The whole blood samples will remain at room temperature until clotted (for approximately 30 minutes). Samples will be centrifuged at approximately 1700 x g for about 10 minutes at 4°C. The serum will be transferred into appropriately labeled screw-capped polypropylene tubes and stored at -20°C within 1 hour of collection until shipment for assay. Samples from each individual subject will be stored in a package for that subject.

Samples will be analyzed for AZ concentrations using a validated analytical method in compliance with Pfizer standard operating procedures. Samples may be used for further evaluation of the bioanalytical method. These data will be used for internal exploratory purposes and will not be included in the clinical report.

7.3.2. Plasma for Analysis of Chloroquine and Desethyl-Chloroquine

Blood samples (4 mL) to provide a minimum of 2 mL plasma for PK analysis of CQ and desethyl-CQ will be collected into appropriately labeled tubes containing K3EDTA at protocol-specified times (See Section 6.1). All efforts will be made to obtain the PK samples at the exact nominal time relative to dosing. However, samples obtained within 10% of the nominal time (eg, within 6 minutes of a 60 minute sample) from dosing will not be captured as a protocol deviation, as long as the exact time of the sample collection is noted on the source document and data collection tool (eg, CRF).

090177e1811d3ccb\Approved\Approved On: 19-Feb-2010 20:59

Specimens will be centrifuged at approximately 1700 x g for about 10 minutes at 4°C. The plasma will be transferred into appropriately labeled screw-capped polypropylene tubes and stored at -20°C within 1 hour of collection until shipment for assay. Samples from each individual subject will be stored as a package for that subject.

Samples will be analyzed for determination of CQ and desethyl-CQ concentrations using a validated analytical method in compliance with Pfizer standard operating procedures. Samples may be used for further evaluation of the bioanalytical method. These data will be used for internal exploratory purposes and will not be included in the clinical report.

7.3.3. Shipment of Pharmacokinetic Samples

The shipment address and assay lab contact information will be provided to the investigator site prior to initiation of the study.

8. ADVERSE EVENT REPORTING

8.1. Adverse Events

All observed or volunteered adverse events regardless of treatment group or suspected causal relationship to the investigational product(s) will be reported as described in the following sections.

For all adverse events, the investigator must pursue and obtain information adequate both to determine the outcome of the adverse event and to assess whether it meets the criteria for classification as a serious adverse event requiring immediate notification to Pfizer or its designated representative. For all adverse events, sufficient information should be obtained by the investigator to determine the causality of the adverse event. The investigator is required to assess causality. For adverse events with a causal relationship to the investigational product, follow-up by the investigator is required until the event or its sequelae resolve or stabilize at a level acceptable to the investigator, and Pfizer concurs with that assessment.

8.2. Reporting Period

For serious adverse events, the reporting period to Pfizer or its designated representative begins from the time that the subject provides informed consent, which is obtained prior to the subject's participation in the study, ie, prior to undergoing any study-related procedure and/or receiving investigational product, **through Day 42 and including 39 calendar days after the last administration of the investigational product, or until one month after the delivery or the termination of pregnancy (which ever is later).** If pregnancy outcome meets the classification as a serious adverse event, it will be reported accordingly as serous adverse event (refer to Section 8.9 for details). Any serious adverse event occurring any time after the reporting period must be promptly reported if a causal relationship to investigational product is suspected.

090177e1811d3ccb\Approved\Approved On: 19-Feb-2010 20:59

Adverse events (serious and non-serious) should be recorded on the CRF from the time the subject has taken at least one dose of study treatment through last subject visit.

8.3. Definition of an Adverse Event

An adverse event is any untoward medical occurrence in a clinical investigation subject administered a product or medical device; the event need not necessarily have a causal relationship with the treatment or usage. Examples of adverse events include but are not limited to:

- Abnormal test findings;
- Clinically significant symptoms and signs;
- Changes in physical examination findings;
- Hypersensitivity;
- Progression/worsening of underlying disease.

Additionally, they may include the signs or symptoms resulting from:

- Drug overdose;
- Drug withdrawal;
- Drug abuse;
- Drug misuse;
- Drug interactions;
- Drug dependency;
- Extravasation.

8.4. Abnormal Test Findings

The criteria for determining whether an abnormal objective test finding should be reported as an adverse event are as follows:

- Test result is associated with accompanying symptoms; and/or
- Test result requires additional diagnostic testing or medical/surgical intervention; and/or

090177e1811d3ccb\Approved\Approved On: 19-Feb-2010 20:59

- Test result leads to a change in study dosing or discontinuation from the study, significant additional concomitant drug treatment, or other therapy; and/or
- Test result is considered to be an adverse event by the investigator or sponsor.

Merely repeating an abnormal test, in the absence of any of the above conditions, does not constitute an adverse event. Any abnormal test result that is determined to be an error does not require reporting as an adverse event.

8.5. Serious Adverse Events

A serious adverse event or serious adverse drug reaction is any untoward medical occurrence at any dose **for mothers or fetus** that:

- Results in death;
- Is life-threatening (immediate risk of death);
- Requires inpatient hospitalization or prolongation of existing hospitalization;
- Results in persistent or significant disability/incapacity;
- Results in congenital anomaly/birth defect.

Medical and scientific judgment should be exercised in determining whether an event is an important medical event. An important medical event may not be immediately life-threatening and/or result in death or hospitalization. However, if it is determined that the event may jeopardize the subject and/or may require intervention to prevent one of the other adverse event outcomes, the important medical event should be reported as serious.

Examples of such events are intensive treatment in an emergency room or at home for allergic bronchospasm; blood dyscrasias or convulsions that do not result in hospitalization; or development of drug dependency or drug abuse.

8.6. Hospitalization

Adverse events reported from studies associated with hospitalization or prolongations of hospitalization are considered serious. Any initial admission (even if less than 24 hours) to a healthcare facility meets these criteria. Admission also includes transfer within the hospital to an acute/intensive care unit (eg, from the psychiatric wing to a medical floor, medical floor to a coronary care unit, neurological floor to a tuberculosis unit).

Hospitalization does not include the following:

- Rehabilitation facilities;
- Hospice facilities;

090177e1811d3ccb\Approved\Approved On: 19-Feb-2010 20:59

- Respite care (eg, caregiver relief);
- Skilled nursing facilities;
- Nursing homes;
- Routine emergency room admissions;
- Same day surgeries (as outpatient/same day/ambulatory procedures).

Hospitalization or prolongation of hospitalization in the absence of a precipitating, clinical adverse event is not in itself a serious adverse event. Examples include:

- Admission for treatment of a preexisting condition not associated with the development of a new adverse event or with a worsening of the preexisting condition (eg, for work-up of persistent pre-treatment lab abnormality);
- Social admission (eg, subject has no place to sleep);
- Administrative admission (eg, for yearly physical exam);
- Protocol-specified admission during a study (eg, for a procedure required by the study protocol);
- Optional admission not associated with a precipitating clinical adverse event (eg, for elective cosmetic surgery);
- Pre-planned treatments or surgical procedures should be noted in the baseline documentation for the entire protocol and/or for the individual subject.

Diagnostic and therapeutic non-invasive and invasive procedures, such as surgery, should not be reported as adverse events. However, the medical condition for which the procedure was performed should be reported if it meets the definition of an adverse event. For example, an acute appendicitis that begins during the adverse event reporting period should be reported as the adverse event, and the resulting appendectomy should be recorded as treatment of the adverse event.

090177e1811d3ccb\Approved\Approved On: 19-Feb-2010 20:59

8.7. Severity Assessment

Table 3. Severity Assessment

If required on the adverse event case report forms, the investigator will use the adjectives MILD, MODERATE, or SEVERE to describe the maximum intensity of the adverse event. For purposes of consistency, these intensity grades are defined as follows:	
MILD	Does not interfere with subject's usual function.
MODERATE	Interferes to some extent with subject's usual function.
SEVERE	Interferes significantly with subject's usual function.

Note the distinction between the severity and the seriousness of an adverse event. A severe event is not necessarily a serious event. For example, a headache may be severe (interferes significantly with subject's usual function) but would not be classified as serious unless it met one of the criteria for serious adverse events, listed above.

8.8. Causality Assessment

The investigator's assessment of causality must be provided for all adverse events (serious and non-serious); the investigator must record the causal relationship in the CRF, as appropriate, and report such an assessment in accordance with the serious adverse reporting requirements if applicable. An investigator's causality assessment is the determination of whether there exists a reasonable possibility that the investigational product caused or contributed to an adverse event. If the investigator does not know whether or not investigational product caused the event, then the event will be handled as "related to investigational product" for reporting purposes, as defined by the Sponsor (See Section on Reporting Requirements). If the investigator's causality assessment is "unknown but not related to investigational product", this should be clearly documented on study records.

In addition, if the investigator determines a serious adverse event is associated with study procedures, the investigator must record this causal relationship in the source documents and CRF, as appropriate, and report such an assessment in accordance with the serious adverse event reporting requirements, if applicable.

8.9. Exposure During Pregnancy

This is a study exclusively in pregnant women participants. Enrolled subjects will be asymptomatic pregnant women who will receive study drugs for preventive treatment of malaria. Since this is the first time AZ and CQ combination will be administered to pregnant women, all subjects will be followed up for EIU safety assessments following delivery or termination of pregnancy. The reason(s) for an induced abortion should be specified. All pregnancy-related AEs through Day 42 following the first dose of AZCQ and the pregnancy outcomes will be recorded on CRF pages as required clinical study data. Since all the information generally included on an EIU form will be collected on CRF paper, a separate EIU form is not required.

090177e1811d3ccb\Approved\Approved On: 19-Feb-2010 20:59

If the outcome of the pregnancy meets the criteria for immediate classification as a serious adverse event (ie, spontaneous abortion, stillbirth, neonatal death, or congenital anomaly [including that in an aborted fetus, stillbirth or neonatal death]), the investigator should follow the procedures for reporting serious adverse events.

In the case of a live birth, the "normality" of the newborn can be assessed at the time of birth (ie, no minimum follow-up period of a presumably normal infant is required before an Exposure in Utero Form can be completed). The "normality" of an aborted fetus can be assessed by gross visual inspection, unless pre-abortion test findings are suggestive of a congenital anomaly.

Additional information about pregnancy outcomes that are classified as serious adverse events follows:

- "Spontaneous abortion" includes miscarriage and missed abortion.
- All neonatal deaths that occur within 1 month of birth should be reported, without regard to causality, as serious adverse events. In addition, any infant death after 1 month that the investigator assesses as possibly related to the exposure during pregnancy to the investigational medication should be reported.

Additional information regarding the EIU may be requested by the investigator. Further follow-up of birth outcomes will be handled on a case-by-case basis (eg, follow-up on preterm infants to identify developmental delays). In the case of paternal exposure, the investigator must obtain permission from the subject's partner in order to conduct any follow-up or collect any information.

8.10. Withdrawal Due to Adverse Events (See also Section on Subject Withdrawal)

Withdrawal due to adverse event should be distinguished from withdrawal due to insufficient response, according to the definition of adverse event noted earlier, and recorded on the appropriate adverse event CRF page.

When a subject withdraws due to a serious adverse event, the serious adverse event must be reported in accordance with the reporting requirements defined below.

Unless a subject withdraws consent or leaves the town/village, she may be withdrawn from the study treatment but should continue in the study as we must follow them up for pregnancy outcome.

8.11. Eliciting Adverse Event Information

The investigator is to report all directly observed adverse events and all adverse events spontaneously reported by the study subject. In addition, each study subject will be questioned about adverse events.

090177e1811d3ccb\Approved\Approved On: 19-Feb-2010 20:59

8.12. Reporting Requirements

Each adverse event is to be assessed to determine if it meets the criteria for serious adverse events. If a serious adverse event occurs, expedited reporting will follow local and international regulations, as appropriate.

8.12.1. Serious Adverse Event Reporting Requirements

If a serious adverse event occurs, Pfizer is to be notified within 24 hours of awareness of the event by the investigator. In particular, if the serious adverse event is fatal or life-threatening, notification to Pfizer must be made immediately, irrespective of the extent of available adverse event information. This timeframe also applies to additional new information (follow-up) on previously forwarded serious adverse event reports as well as to the initial and follow-up reporting of Exposure during pregnancy cases.

This timeframe of reporting within 24 hours also applies for the reporting of any medical device complaint.

In the rare event that the investigator does not become aware of the occurrence of a serious adverse event immediately (eg, if an outpatient study subject initially seeks treatment elsewhere), the investigator is to report the event within 24 hours after learning of it and document the time of his/her first awareness of the adverse event.

For all serious adverse events, the investigator is obligated to pursue and provide information to Pfizer in accordance with the timeframes for reporting specified above. In addition, an investigator may be requested by Pfizer to obtain specific additional follow-up information in an expedited fashion. This information may be more detailed than that captured on the adverse event case report form. In general, this will include a description of the adverse event in sufficient detail to allow for a complete medical assessment of the case and independent determination of possible causality. Information on other possible causes of the event, such as concomitant medications and illnesses must be provided. In the case of a subject death, a summary of available autopsy findings must be submitted as soon as possible to Pfizer or its designated representative.

In addition, the Investigator will follow all applicable local and national safety reporting guidelines and regulations for notification to their local ethics and regulatory bodies.

8.12.2. Non-Serious Adverse Event Reporting Requirements

All adverse events will be reported on the adverse event page(s) of the CRF. It should be noted that the form for collection of serious adverse event information is not the same as the adverse event CRF. Where the same data are collected, the forms must be completed in a consistent manner. For example, the same adverse event term should be used on both forms. Adverse events should be reported using concise medical terminology on the CRFs as well as on the form for collection of serious adverse event information.

090177e1811d3ccb\Approved\Approved On: 19-Feb-2010 20:59

8.12.3. Medical Device Complaint Reporting Requirements

Not applicable.

8.12.4. Sponsor Reporting Requirements to Regulatory Authorities

Adverse events reporting, including suspected serious unexpected adverse reactions, will be carried out in accordance with applicable local regulations.

9. DATA ANALYSIS/STATISTICAL METHODS

Detailed methodology for summary and statistical analyses of the data collected in this trial will be documented in an Analysis Plan, which will be dated and maintained by the Sponsor. This document may modify the plans outlined in the protocol; however, any major modifications of the primary endpoint definition and/or its analysis will also be reflected in a protocol amendment.

An early analysis of the data will be performed on parasite related endpoints (up to Day 42), including the primary, when all subjects achieve their Day 42 visit (ie, 42 days of follow-up post first dose), or withdraw from the study early.

9.1. Sample Size Determination

There will be approximately 166 subjects in the study. This will be a single arm, non-comparative estimation study and will not be powered for formal hypothesis testing. The sample size will yield a half width of a 2-sided 95% confidence interval to be ≤5 percentage points with probability=0.80 for the primary efficacy endpoint, using a large sample normal approximation to the binomial provided the true underlying incidence is ≥95%, and assuming approximately 10% will drop out.

9.2. Parasitological Response Analysis

Three study populations will be defined as follows.

- Intent-To-Treat (ITT): ITT is defined as all subjects who receive at least one dose of study medication and who have baseline blood smears positive for *Plasmodium falciparum* monoinfection, asexual parasitemia.
- Modified Intent-To-Treat (MITT): MITT is a subset of the ITT Population who has *Plasmodium falciparum* monoinfection (confirmed by microscopy) parasite counts in the range of 80-100,000/μL on thick blood smears.
- Per Protocol (PP): PP is a subset of MITT subjects who receive all 3 days of study medication.

090177e1811d3ccb\Approved\Approved On: 19-Feb-2010 20:59

9.2.1. Analysis of Primary Endpoint

The proportion of subjects with parasitological response (PCR corrected) at Day 28 post first dose of study medication will be estimated for the primary endpoint using the MITT and PP subject populations. The proportion will be estimated from the Kaplan-Meier curve based on the time to the first occurrence of parasitological failure (PCR corrected). Use of the Kaplan-Meier curve/product limit estimator is recommended by WHO[8] and has been used in analyzing primary efficacy endpoints in late stage antimalarial treatment clinical trials.[92-94]

A subject will be a parasitological responder if she has a zero parasite count on the Day 7 visit without subsequent recrudescence (PCR corrected) through the day of consideration, otherwise she is a parasitological failure. In addition, subjects who withdraw from the study treatment during the first three days due to development of malaria symptoms and are administered antimalarial standard of care treatment for malaria in pregnancy, will be considered parasitological failures. The time (number of days) to the first occurrence of parasitological failure will be the analysis variable. The time in days is defined as [date of the parasitological failure event – date of the first dose of study medication + 1].

Subjects who do not experience the defined event (parasitological failure) will be censored as follows:

- Subjects who withdraw from the study early due to any reason will be censored on the day of the last available blood smear measurement;
- Subjects who continue on in the study but have all remaining blood smears missing from a given point in time onward will be censored on the day of the last available smear;
- Subjects completing the Day 42 visit post first dose of study medication will be censored on the day of the last available blood smear measurement;
- Subjects receiving any concomitant antimalarial for reinfection (PCR corrected) or in the absence of parasitemia will be censored at the visit date if the antimalarial is given after collection of the blood smear on that day. Otherwise the subject will be censored on the day of the last available previous blood smear measurement;
- Subjects who are lost to follow-up without any follow-up blood smears will have a censored duration of 1 day.

The proportion of subjects with parasitological response (PCR corrected) at Day 28 (primary) will be estimated from the Kaplan-Meier curve and its standard error estimated by the Greenwood formula. If less than 100%, a two-sided 95% confidence interval for the estimated proportion of subjects with parasitological response will be computed.

090177e1811d3ccb\Approved\Approved On: 19-Feb-2010 20:59

Additional sensitivity analyses will also be performed using the MITT and PP subject populations. The sensitivity analyses will consider premature discontinuation due to any reason as parasitological failure, if not already a parasitological failure due to a defined event. The same statistical methods will again be used to estimate the proportion of subjects with parasitological response (PCR corrected) at Day 28.

9.2.2. Analysis of Secondary Endpoints

The secondary parasitological response endpoints will be analyzed in the same manner as the primary endpoint, and will use the ITT, MITT, and PP subject populations. These analyses will be done using both PCR corrected and uncorrected results, and will also include the sensitivity analyses.

For PCR corrected, censoring will be done in the same manner as for the primary endpoint. For PCR uncorrected, censoring will be as follows for subjects who do not experience parasitological failure:

- Subjects who withdraw from the study early due to any reason will be censored on the day of the last available blood smear measurement;
- Subjects who continue on in the study but have all remaining blood smears missing from a given point in time onward will be censored on the day of the last available smear;
- Subjects completing the Day 42 visit post first dose of study medication will be censored on the day of the last available blood smear measurement;
- Subjects receiving any concomitant antimalarial in the absence of parasitemia will be censored at the visit date if the antimalarial is given after collection of the blood smear on that day. Otherwise the subject will be censored on the day of the last available previous blood smear measurement;
- Subjects who are lost to follow-up without any follow-up blood smears will have a censored duration of 1 day.

For parasite counts, descriptive statistics (N, mean, SD, standard error, median, minimum and maximum) will be provided at each time point on actual counts.

9.3. Safety Analysis

Adverse events, history of concomitant treatments, hemoglobin, temperature, and the EIU safety assessment will be recorded for each subject during the study according to the schedule of assessments. Standard safety reporting tables will summarize and list the safety data.

090177e1811d3ccb\Approved\Approved On: 19-Feb-2010 20:59

Each adverse event will be counted once according to the date of onset. If the adverse event onset was prior to the first dose of study drug and the event does not increase in severity after initiation of study drug, the adverse event is then considered to be a pre-treatment adverse event and will not be counted in the treatment-emergent adverse event incidence tables. If the onset is prior to the first dose of study drug and the severity increases thereafter, the event is counted as a treatment-emergent adverse event. An adverse event with onset after the first dose of study drug will be counted as a treatment-emergent adverse event. This rule is consistent with the treatment-emergent signs and symptoms (TESS) convention for counting adverse events.

9.4. Pharmacokinetic Analysis

The PK concentration population is defined as all subjects who have received at least 1 dose and have at least 1 blood sample collected for PK analysis prior to subject withdraw from the study, if applicable.

Individual concentrations of AZ, CQ, and desethyl-CQ will be summarized via descriptive statistics by nominal time and listed by nominal time and actual time. Actual PK sampling times will be used in the derivation of PK parameters. As data permitting, all PK concentration data may contribute to characterizing the PK of AZCQ using a population PK approach with pooled data from other studies and will be reported separately. Actual PK sampling times will be used in the derivation of PK parameters.

9.5. Interim Analysis

None.

9.6. Independent Data Monitoring Committee (IDMC)

An IDMC external from Pfizer will oversee the study for safety aspects, and will review emerging safety data on an ongoing basis. The full composition of the IDMC and the guidelines for oversight of this study will be specified in the IDMC charter.

10. QUALITY CONTROL AND QUALITY ASSURANCE

During study conduct, Pfizer or its agent will conduct periodic monitoring visits to ensure that the protocol and GCPs are being followed. The monitors may review source documents to confirm that the data recorded on CRFs is accurate. The investigator and institution will allow Pfizer monitors or its agents and appropriate regulatory authorities direct access to source documents to perform this verification.

The study site may be subject to review by the Institutional Review Board (IRB)/Independent Ethics Committee (IEC), and/or to quality assurance audits performed by Pfizer, or companies working with or on behalf of Pfizer, and/or to inspection by appropriate regulatory authorities.

090177e1811d3ccb\Approved\Approved On: 19-Feb-2010 20:59

It is important that the investigator(s) and their relevant personnel are available during the monitoring visits and possible audits or inspections and that sufficient time is devoted to the process.

11. DATA HANDLING AND RECORD KEEPING

11.1. Case Report Forms/Electronic Data Record

As used in this protocol, the term Case Report Form (CRF) should be understood to refer to either a paper form or an electronic data record or both, depending on the data collection method used in this study.

A CRF is required and should be completed for each included subject. The completed original CRFs are the sole property of Pfizer and should not be made available in any form to third parties, except for authorized representatives of Pfizer or appropriate regulatory authorities, without written permission from Pfizer.

The investigator has ultimate responsibility for the collection and reporting of all clinical, safety and laboratory data entered on the CRFs and any other data collection forms (source documents) and ensuring that they are accurate, authentic / original, attributable, complete, consistent, legible, timely (contemporaneous), enduring and available when required. The CRFs must be signed by the investigator or by an authorized staff member to attest that the data contained on the CRFs is true. Any corrections to entries made in the CRFs, source documents must be dated, initialed and explained (if necessary) and should not obscure the original entry.

In most cases, the source documents are the hospital's or the physician's subject chart. In these cases data collected on the CRFs must match the data in those charts.

In some cases, the CRF, or part of the CRF, may also serve as source documents. In these cases, a document should be available at the investigator’s site as well as at Pfizer and clearly identify those data that will be recorded in the CRF, and for which the CRF will stand as the source document.

11.2. Record Retention

To enable evaluations and/or audits from regulatory authorities or Pfizer, the investigator agrees to keep records, including the identity of all participating subjects (sufficient information to link records, eg, CRFs and hospital records), all original signed informed consent forms, copies of all CRFs, serious adverse event forms, source documents, and detailed records of treatment disposition, and adequate documentation of relevant correspondence (eg, letters, meeting minutes, telephone calls reports). The records should be retained by the investigator according to ICH, local regulations, or as specified in the Clinical Study Agreement, whichever is longer.

090177e1811d3ccb\Approved\Approved On: 19-Feb-2010 20:59

If the investigator becomes unable for any reason to continue to retain study records for the required period (eg, retirement, relocation), Pfizer should be prospectively notified. The study records must be transferred to a designee acceptable to Pfizer, such as another investigator, another institution, or to an independent third party arranged by Pfizer. The investigator must obtain Pfizer's written permission before disposing of any records, even if retention requirements have been met.

12. ETHICS

12.1. Institutional Review Board (IRB)/Independent Ethics Committee (IEC)

The IRB/IEC of London School of Hygiene and Tropical Medicine (LSHTM) is planned to be used in this study in addition to local IRB/IEC. Study related documentation will be reviewed by the LSHTM IRB/IEC and the local IRB/IEC. No subjects will be enrolled in the study until the approvals from both LSHTM IRB/IEC and local IRB/IEC are received. It is the responsibility of the investigator to have prospective approval of the study protocol, protocol amendments, informed consent forms, and other relevant documents, eg, recruitment advertisements, if applicable, from the IRB/IEC. All correspondence with the IRB/IEC should be retained in the Investigator File. Copies of IRB/IEC approvals should be forwarded to Pfizer.

The only circumstance in which an amendment may be initiated prior to IRB/IEC approval is where the change is necessary to eliminate apparent immediate hazards to the subjects. In that event, the investigator must notify the IRB/IEC and Pfizer in writing immediately after the implementation.

12.2. Ethical Conduct of the Study

The study will be conducted in accordance with the Declaration of Helsinki on Ethical Principles for Medical Research Involving Human Subjects, adopted by the General Assembly of the World Medical Association (1996).

In addition, the study will be conducted in accordance with the protocol, the International Conference on Harmonization guideline on Good Clinical Practice, and applicable local regulatory requirements and laws.

12.3. Subject Information and Consent

All parties will ensure protection of subject personal data and will not include subject names on any sponsor forms, reports, publications, or in any other disclosures, except where required by laws. In case of data transfer, Pfizer will maintain high standards of confidentiality and protection of subject personal data.

The informed consent and assent form must be in compliance with ICH GCP, local regulatory requirements, and legal requirements. Assent will be obtained from subjects <18 years of age. Local IECs/IRBs will determine the age at which assent must be obtained from the subject.

090177e1811d3ccb\Approved\Approved On: 19-Feb-2010 20:59

The informed consent and assent form used in this study, and any changes made during the course of the study, must be prospectively approved by both the IRB/IEC and Pfizer before use.

The investigator must ensure that each study subject, or his/her legally acceptable representative, is fully informed about the nature and objectives of the study and possible risks associated with participation. The investigator, or a person designated by the investigator, will obtain written informed consent from each subject or the subject's legally acceptable representative before any study-specific activity is performed. The investigator will retain the original of each subject's signed consent form.

12.4. Reporting of Safety Issues and Serious Breaches of the Protocol or ICH GCP

In the event of any prohibition or restriction imposed (ie, clinical hold) by an applicable Competent Authority in any area of the World, or if the investigator is aware of any new information which might influence the evaluation of the benefits and risks of the investigational product, Pfizer should be informed immediately.

In addition, the investigator will inform Pfizer immediately of any urgent safety measures taken by the investigator to protect the study subjects against any immediate hazard, and of any serious breaches of this protocol or of ICH GCP that the investigator becomes aware of.

13. DEFINITION OF END OF TRIAL

13.1. End of Trial in all Participating Countries

End of Trial in all participating countries is defined as Last Subject Last Visit which may have been determined by the point at which the dose escalation and study stopping rules have been met

14. SPONSOR DISCONTINUATION CRITERIA

Premature termination of this study may occur because of a regulatory authority decision, change in opinion of the IRB/IEC, drug safety problems, or at the discretion of Pfizer. In addition, Pfizer retains the right to discontinue development of AZCQ at any time.

If a study is prematurely terminated or discontinued, Pfizer will promptly notify the investigator. After notification, the investigator must contact all participating subjects and the hospital pharmacy (if applicable) within 30 days. As directed by Pfizer, all study materials must be collected and all CRFs completed to the greatest extent possible.

15. PUBLICATION OF STUDY RESULTS

Publication of study results is discussed in the Clinical Study Agreement.

090177e1811d3ccb\Approved\Approved On: 19-Feb-2010 20:59

15.1. Communication of results by Pfizer

Pfizer fulfils its commitment to publicly disclose the results of studies through posting the results of this study on ClinicalStudyResults.org. Pfizer posts the results of studies that fall into either of the following categories:

- Studies that Pfizer registered on www.clinicaltrials.gov, (ClinicalTrials.gov) regardless of the reason for registration; OR
- All other studies for which the results have scientific or medical importance as determined by Pfizer.

For studies involving a Pfizer product, the timing of the posting depends on whether the Pfizer product is approved for marketing in any country at the time the study is completed:

- For studies involving products already approved in any country and for studies that do not involve a Pfizer product, Pfizer posts results within one year after study completion, defined as Last Subject, Last Visit (LSLV);
- For studies involving products that are not yet approved in any country, Pfizer posts the results of already-completed studies within one year after the first regulatory approval of the product;
- For studies involving products whose drug development is discontinued before approval, Pfizer posts the results within one year after such discontinuation.

Pfizer's posting on ClinicalStudyResults.org includes the following elements:

- Protocol title, study phase, and indication;
- A link to approved product labeling, if applicable;
- The synopsis of study results;
- Citations of known study publications;
- Legal disclaimer.

The study results synopsis posted on ClinicalStudyResults.org (called the PhRMA website synopsis) uses the format established by the ICH-E3 Clinical Study Report (CSR) Synopsis. If posting of study results to ClinicalStudyResults.org jeopardizes a planned publication of the study results, a Pending Full Publication notice is substituted for the synopsis until the study results publication has issued or two years have elapsed, whichever occurs first.

Pfizer posts citations only for publications that are accessible in recognized (searchable) publication databases. Single-centre results publications for a multi-centre study are generally not posted because they may not accurately reflect the results of the study.

090177e1811d3ccb\Approved\Approved On: 19-Feb-2010 20:59

15.2. Publications by Investigators

Pfizer has no objection to publication by Investigator of any information collected or generated by Investigator, whether or not the results are favorable to the Investigational Drug. However, to ensure against inadvertent disclosure of Confidential Information or unprotected Inventions, Investigator will provide Pfizer an opportunity to review any proposed publication or other type of disclosure before it is submitted or otherwise disclosed.

Investigator will provide manuscripts, abstracts, or the full text of any other intended disclosure (poster presentation, invited speaker or guest lecturer presentation, etc) to Pfizer at least 30 days before they are submitted for publication or otherwise disclosed. If any patent action is required to protect intellectual property rights, Investigator agrees to delay the disclosure for a period not to exceed an additional 60 days.

Investigator will, on request, remove any previously undisclosed Confidential Information (other than the Study results themselves) before disclosure.

If the Study is part of a multi-centre study, Investigator agrees that the first publication is to be a joint publication covering all centers. However, if a joint manuscript has not been submitted for publication within 12 months of completion or termination of the Study at all participating sites, Investigator is free to publish separately, subject to the other requirements of this Section.

For all publications relating to the Study, Institution will comply with recognized ethical standards concerning publications and authorship, including Section II - "Ethical Considerations in the Conduct and Reporting of Research" of the Uniform Requirements for Manuscripts Submitted to Biomedical Journals, http://www.icmje.org/index.html#authorship, established by the International Committee of Medical Journal Editors.

Publication of study results is also provided for in the Clinical Study Agreement between Pfizer and the institution. In this section entitled Publications by Investigators, the defined terms shall have the meanings given to them in the Clinical Study Agreement.

090177e1811d3ccb\Approved\Approved On: 19-Feb-2010 20:59

16. REFERENCES

1. Winstanley, P., Modern chemotherapeutic options for malaria. The Lancet Infectious Diseases, 2001. 1(4): p. 242-250.

2. Greenwood, B. and T. Mutabingwa, Malaria in 2002. Nature, 2002. 415(6872): p. 670-672.

3. WHO, WORLD MALARIA REPORT 2009 2009. http://www.who.int/malaria/publications/atoz/9789241563901/en/index.html

4. Chico M, e.a., Azithromycin-chloroquine and the intermittent preventive treatment of malaria in pregnancy. Malaria Journal, 2008. 7: p. 255.

5. World Health Orgnization (WHO). Malaria in Pregnancy. Retrieved on January, 2010 from http://www.who.int/malaria/high_risk_groups/pregnancy/en/index.html

6. WHO, A Strategic Framework for Malaria Prevention and Control During Pregnancy in the Africa Region. Vol. AFR/MAL/04/01. 2004: World Health Organization Regional Office for Africa.

7. Kayentao K, K.M., Newman RD, et al. , Comparison of intermittent preventive treatment with chemoprophylaxis for the prevention of malaria during pregnancy in Mali. J Infect Dis, 2005. 191 (1): p. 109-16.

8. WHO, Assessment and Monitoring of antimalarial drug efficacy for the treatment of uncomplicated falciparum malaria. Vol. WHO/HTM/RBM/2003.50. 2003, Geneva: World Health Organization.

9. Roper, C., et al., Antifolate antimalarial resistance in southeast Africa: a population-based analysis. Lancet, 2003. 361(9364): p. 1174-81.

10. Briand, V.r., et al., Intermittent Treatment for the Prevention of Malaria during Pregnancy in Benin: A Randomized, Openâ€□Label Equivalence Trial Comparing Sulfadoxineâ€□Pyrimethamine with Mefloquine. The Journal of Infectious Diseases, 2009. 200(6): p. 991-1001.

11. Smoak, B.L., et al., The effects of inadvertent exposure of mefloquine chemoprophylaxis on pregnancy outcomes and infants of US Army servicewomen. J Infect Dis, 1997. 176(3): p. 831-3.

12. Nosten, F., et al., The effects of mefloquine treatment in pregnancy. Clin Infect Dis, 1999. 28(4): p. 808-15.

13. Clerk, C.A., et al., A Randomized, Controlled Trial of Intermittent Preventive Treatment with Sulfadoxine-Pyrimethamine, Amodiaquine, or the Combination in Pregnant Women in Ghana. J Infect Dis, 2008.

090177e1811d3ccb\Approved\Approved On: 19-Feb-2010 20:59

14. Tagbor, H., et al., Efficacy, safety, and tolerability of amodiaquine plus sulphadoxine-pyrimethamine used alone or in combination for malaria treatment in pregnancy: a randomised trial. Lancet, 2006. 368(9544): p. 1349-56.

15. Fanello, C.I., et al., Tolerability of amodiaquine and sulphadoxine-pyrimethamine, alone or in combination for the treatment of uncomplicated Plasmodium falciparum malaria in Rwandan adults. Trop Med Int Health, 2006. 11(5): p. 589-96.

16. Vallely A, V.L., Changalucha J, Greenwood B, Chandramohan D Intermittent preventive treatment for malaria in pregnancy in Africa: what's new, what's needed? . Malar J, 2007. 6: p. 16.

17. WHO, Assessment of the Safety of Artemisinin Compounds in Pregnancy. Report of two informal consultations convened by WHO in 2002 (Roll Back Malaria and the UNDP/World Bank/WHO Special Programme for Research and Training in Tropical Diseases). Vol. WHO/CDS/MAL/2003.1094. WHO/RBM/TDR/Artemisinin/03.1. 2003, Geneva: World Health Organization [http://www.who.int/malaria/cmc_upload/0/000/016/323/artemisinin_pregnancy.pdf]

18. Andersen SL, A.A., McGreevy P, et al. , Activity of azithromycin as a blood schizonticide against rodent and human plasmodia in vivo. . American Journal of Tropical Medicine & Hygiene 1995. 52: p. 159-61.

19. Sadiq, S.T., et al., Effects of azithromycin on malariometric indices in The Gambia. Lancet, 1995. 346(8979): p. 881-2.

20. Greenwood, B., et al., Malaria in pregnancy: priorities for research. Lancet Infect Dis, 2007. 7(2): p. 169-74.

21. Kiddugavu, M.G., et al., Effectiveness of syphilis treatment using azithromycin and/or benzathine penicillin in Rakai, Uganda. Sex Transm Dis, 2005. 32(1): p. 1-6.

22. Riedner, G., et al., Single-dose azithromycin versus penicillin G benzathine for the treatment of early syphilis. N Engl J Med, 2005. 353(12): p. 1236-44.

23. Gray, R.H., et al., Randomized trial of presumptive sexually transmitted disease therapy during pregnancy in Rakai, Uganda. American Journal of Obstetrics and Gynecology, 2001. 185(5): p. 1209-1217.

24. Kaul, R., et al., Monthly antibiotic chemoprophylaxis and incidence of sexually transmitted infections and HIV-1 infection in Kenyan sex workers: a randomized controlled trial. JAMA, 2004. 291(21): p. 2555-62.

25. Morency, A.M. and E. Bujold, The effect of second-trimester antibiotic therapy on the rate of preterm birth. J Obstet Gynaecol Can, 2007. 29(1): p. 35-44.

090177e1811d3ccb\Approved\Approved On: 19-Feb-2010 20:59

26. Girard, A.E., et al., Pharmacokinetic and in vivo studies with azithromycin (CP-62,993), a new macrolide with an extended half-life and excellent tissue distribution. Antimicrob Agents Chemother, 1987. 31(12): p. 1948-54.

27. Dunn CJ and Barradell LB, Azithromycin. A review of its pharmacological properties and use as 3-day therapy in respiratory tract infections. Drugs, 1996. 51(3) p. 483-505.

28. Ballow, C.H. and G.W. Amsden, Azithromycin: the first azalide antibiotic. Ann Pharmacother, 1992. 26(10): p. 1253-61.

29. Peters, D.H., H.A. Friedel, and D. McTavish, Azithromycin. A review of its antimicrobial activity, pharmacokinetic properties and clinical efficacy. Drugs, 1992. 44(5): p. 750-99.

30. Sidhu AB, et al., In vitro efficacy, resistance selection, and structural modeling studies implicate the malarial parasite apicoplast as the target of azithromycin. J Biol Chem, 2007. 282(4): p. 2494-504.

31. WHO, Sexually transmitted and other reproductive tract infections: Guide to essential practice. Integrating STI/RTI Care for Reproductive Health. 2005, Geneva: Department of Reproductive Health and Research, World Health Organization.

32. Araujo, F.G., D.R. Guptill, and J.S. Remington, Azithromycin, a macrolide antibiotic with potent activity against Toxoplasma gondii. Antimicrob Agents Chemother, 1988. 32(5): p. 755-7.

33. Crouch, A.A., et al., Sensitivity in vitro of Giardia intestinalis to dyadic combinations of azithromycin, doxycycline, mefloquine, tinidazole and furazolidone. Trans R Soc Trop Med Hyg, 1990. 84(2): p. 246-8.

34. Ravdin, J.I. and J. Skilogiannis, In vitro susceptibilities of Entamoeba histolytica to azithromycin, CP-63,956, erythromycin, and metronidazole. Antimicrob Agents Chemother, 1989. 33(6): p. 960-2.

35. Krause, P.J., et al., Atovaquone and azithromycin for the treatment of babesiosis. N Engl J Med, 2000. 343(20): p. 1454-8.

36. Noedl H, W.W., Krudsood S, et al. , Antimalarial activity of azithromycin, artemisinin and dihydroartemisinin in fresh isolates of Plasmodium falciparum in Thailand. . Acta Tropica 2001. 80: p. 39-44.

37. Sidhu ABS, et al., Identification of a novel mutation in the L4 plastid ribosomal protein in P. falciparum azithromycin-resistant lines, in 54th Annual Meeting of the American Society of Topical Medicine and Hygiene. 2005, Albert Einstein College of Medicine and Pfizer Inc: Washington DC.

090177e1811d3ccb\Approved\Approved On: 19-Feb-2010 20:59

38. Gingras, B. and J. Jensen, Activity of azithromycin or erythromycin in combination with antimalarial drugs against multidrug-resistant Plasmodium falciparum in vitro. Am J Trop Med. Hygiene, 1992. 47: p. 378-382.

39. Gingras, B. and J. Jensen, Antimalarial activity of azithromycin and erythromycin against Plasmodium burghei. Am J Trop Med, 1993. 49: p. 101-105.

40. Yeo AE, R.K., Increased antimalarial activity of azithromycin during prolonged exposure of Plasmodium falciparum in vitro. Int J Parasitol 1995. 25: p. 531-2.

41. Biswas, S., In vitro antimalarial activity of azithromycin against chloroquine sensitive and chloroquine resistant Plasmodium falciparum. Journal of Postgraduate Medicine, 2001. 47 p. 240-3.

42. Duff, P., Antibiotic selection in obstetric patients. Infect Dis Clin North Am, 1997. 11(1): p. 1-12.

43. Ogasawara, K.K. and T.M. Goodwin, Efficacy of azithromycin in reducing lower genital Ureaplasma urealyticum colonization in women at risk for preterm delivery. J Matern Fetal Med, 1999. 8(1): p. 12-6.

44. Donders, G.G., Treatment of sexually transmitted bacterial diseases in pregnant women. Drugs, 2000. 59(3): p. 477-85.

45. Ramsey, P.S., et al., Maternal and transplacental pharmacokinetics of azithromycin. Am J Obstet Gynecol, 2003. 188(3): p. 714-8.

46. Heikkinen, T., et al., The transplacental transfer of the macrolide antibiotics erythromycin, roxithromycin and azithromycin. BJOG, 2000. 107(6): p. 770-5.

47. Liu, P., et al., Comparative Pharmacokinetics of Azithromycin in Serum and White Blood Cells of Healthy Subjects Receiving a Single-Dose Extended-Release Regimen versus a 3-Day Immediate-Release Regimen. Antimicrob. Agents Chemother., 2007. 51(1): p. 103-109.

48. Taylor WR, et al., Malaria prophylaxis using azithromycin: a double-blind, placebo-controlled trial in Irian Jaya, Indonesia. Clin Infect Dis 1999. 28: p. 74–81.

49. Heppner, D.G., Jr., et al., Randomized, controlled, double-blind trial of daily oral azithromycin in adults for the prophylaxis of Plasmodium vivax malaria in Western Thailand. Am J Trop Med Hyg, 2005. 73(5): p. 842-9.

50. Andersen, S.L., et al., Successful double-blinded, randomized, placebo-controlled field trial of azithromycin and doxycycline as prophylaxis for malaria in western Kenya. Clin Infect Dis, 1998. 26(1): p. 146-50.

090177e1811d3ccb\Approved\Approved On: 19-Feb-2010 20:59

51. Kuschner, R., et al., Azithromycin prophylaxis against a chloroquine resistant strain of Plasmodium falciparum. Lancet, 1994. 343(8910): p. 1396–1397.

52. Anderson, S., et al., Prophylaxis of Plasmodium falciparum malaria with azithromycin administered to volunteers. Ann Intern Med, 1995. 123: p. 771–773.

53. Dunne, MichaelÂ W., et al., A Multicenter Study of Azithromycin, Alone and in Combination with Chloroquine, for the Treatment of Acute Uncomplicated Plasmodium falciparum Malaria in India. The Journal of Infectious Diseases, 2005. 191(10): p. 1582-1588.

54. Pfizer, Trovano®/Zithromax® Compliance Pak. 1998: [http://www.fda.gov/CDER/foi/label/1998/50-762.pdf].

55. Pitsouni, E., et al., Single-dose azithromycin versus erythromycin or amoxicillin for Chlamydia trachomatis infection during pregnancy: a meta-analysis of randomised controlled trials. Int J Antimicrob Agents, 2007. 30(3): p. 213-21.

56. Bar-Oz B, Diav-Citrin, O, Schechtman, S, et al. Pregnancy outcome after gestational exposure to the new macrolides: a prospective multi-center observational study. European J of Obstetrics and Gynecology and Reproductive Biology 2008;141:31-34.

57. Cooper W, Hernandez-Diaz, S, Arbogast PG, et al. Antibiotics potentially used in response to bioterrorism and the risk of major congenital malformations. Pediatric and Perinatal Epidemiology 2008;23:18-28.

58. Kalliani L, Mofolo I, Chaponda M, et al. A randomized controlled pilot trial of azithromycin or astesunate added to sulfadoxine-pryimethamine as treatment for malaria in pregnant women. PLoS ONE. Vol. 2, 2007;e1166. doi:10.1371/journal.pone.0001166.

59. Sidhu, A.B., D. Verdier-Pinard, and D.A. Fidock, Chloroquine resistance in Plasmodium falciparum malaria parasites conferred by pfcrt mutations. Science, 2002. 298(5591): p. 210-3.

60. Fidock DA, et al., Mutations in the P. falciparum digestive vacuole transmembrane protein PfCRT and evidence for their role in chloroquine resistance. Mol Cell, 2000. 6(4): p. 861-71.

61. Reprotox. Website accessed electronically on May, 2006 from http://csi.micromedex.com/DATA/RX/RX1056.HTM?Top=Yes.

62. Udalova et al, from Shepard's Catalog of Teratogenic Agents. Website accessed electronically on May, 2006 from http://csi.micromedex.com/DATA/SH/SH660.HTM?Top=Yes

090177e1811d3ccb\Approved\Approved On: 19-Feb-2010 20:59

63. Walker, B. and Warner, C. (1974). Proceedings: A preliminary investigation of the teratogenic action of chloroquine in the rat. West African J Pharmacol Drug Res 2: 61P-62P.

64. Sharma, A. and Rawat, A.K.(1989) Toxicological Consequences of Chloroquine and Ethanol on the Developing Fetus. Pharmacology Biochemistry & Behavior 34: 77-82.

65. Lee, S., et al., Chloroquine pharmacokinetics in pregnant and nonpregnant women with vivax malaria. European Journal of Clinical Pharmacology, 2008. 64(10): p. 987-992.

66. Denoeud, L., et al., Is chloroquine chemoprophylaxis still effective to prevent low birth weight? Results of a study in Benin. Malar J, 2007. 6: p. 27.

67. Mita, T., et al., Recovery of chloroquine sensitivity and low prevalence of the Plasmodium falciparum chloroquine resistance transporter gene mutation K76T following the discontinuance of chloroquine use in Malawi. Am J Trop Med Hyg, 2003. 68(4): p. 413-5.

68. Takechi, M., et al., Therapeutic efficacy of sulphadoxine/pyrimethamine and susceptibility in vitro of P. falciparum isolates to sulphadoxine-pyremethamine and other antimalarial drugs in Malawian children. Trop Med Int Health, 2001. 6(6): p. 429-34.

69. Djimde, A., et al., A molecular marker for chloroquine-resistant falciparum malaria. N Engl J Med, 2001. 344(4): p. 257-63.

70. Kublin, J.G., et al., Reemergence of chloroquine-sensitive Plasmodium falciparum malaria after cessation of chloroquine use in Malawi. J Infect Dis, 2003. 187(12): p. 1870-5.

71. Laufer, M.K., et al., Return of chloroquine antimalarial efficacy in Malawi. N Engl J Med, 2006. 355(19): p. 1959-66.

72. Hastings, I.M. and M.J. Donnelly, The impact of antimalarial drug resistance mutations on parasite fitness, and its implications for the evolution of resistance. Drug Resist Updat, 2005. 8(1-2): p. 43-50.

73. Laufer, M.K. and C.V. Plowe, Withdrawing antimalarial drugs: impact on parasite resistance and implications for malaria treatment policies. Drug Resist Updat, 2004. 7(4-5): p. 279-88.

74. Walliker, D., P. Hunt, and H. Babiker, Fitness of drug-resistant malaria parasites. Acta Trop, 2005. 94(3): p. 251-9.

75. Mwai, L., et al., Chloroquine resistance before and after its withdrawal in Kenya. Malar J, 2009. 8: p. 106.

090177e1811d3ccb\Approved\Approved On: 19-Feb-2010 20:59

76. Neely, M., et al., Effect of chloroquine on human immunodeficiency virus (HIV) vertical transmission. Afr Health Sci, 2003. 3(2): p. 61-7.

77. Semrau, K., et al., Impact of chloroquine on viral load in breast milk. Trop Med Int Health, 2006. 11(6): p. 800-3.

78. Jaeger, A., Poisonous substances: Quinine and chloroquine. Medicine, 2007. 35: p. 652-653.

79. Sowunmi, A., O. Walker, and L.A. Salako, Pruritus and antimalarial drugs in Africans. Lancet, 1989. 2(8656): p. 213.

80. Mutabingwa, T., L. Villegas, and F. Nosten, Chemoprophylaxis and other protective measures: Preventing pregnancy malaria, in Malaria in Pregnancy: Deadly Parasite, Susceptible Host, P. Duffy and M. Fried, Editors. 2001, Taylor and Francis: London. p. 189-222.

81. Tagbor H, Bruce J, Ord R, Randall A, et al. . Comparison of the therapeutic efficacy of chloroquine and sulphadoxine-pyremethamine in children and pregnant women. Tropical Medicine and International Health 2007;12:1288-1297.

82. Briand V, Denoeund L, Massougbodji, A, et al. Efficacy of intermittent preventive treatment versus chloroquine prophylaxis to prevent malaria during pregnancy in Benin. Journal of Infectious Diseases 2008;198:594-601.

83. Asa O, Onayade, AA, Fatusi, AO. Efficacy of intermittent preventive treatment of malaria with sulphadoxine-pyrimethamine in preventing anaemia in pregnancy among Nigerian women. Matern Child Health J 2008;12:692-698.

84. Law I, Ilett KF, Hackett, P, et al. Transfer of chloroquine and desethylcholorquine across the placenta and into milk in Melanesian mothers. British J of Clinical Pharmacology 1008;65:674-679.

85. Ohrt, C., et al., Assessment of azithromycin in combination with other antimalarial drugs against Plasmodium falciparum in vitro. Antimicrob Agents Chemother, 2002. 46(8): p. 2518-24.

86. Salman, S., S. J. Rogerson, et al. (2009). The pharmacokinetic properties of azithromycin in pregnancy. Antimicrob. Agents Chemother.: AAC.00771-09.

87. Chandra R, L.D., Moran D, Dubhashi N, Sarkar S, Wang C, Cai J, Dunne M, A phase 2, open label, non-comparative trial of Azithromycin 2g plus chloroquine 600 mg base daily for three days for the treatment of uncomplicated Plasmodium falciparum malaria. . 57th ASTMH (American Society of Tropical Medicine and Hygiene) annual meeting, New Orleans, USA, 2008.

090177e1811d3ccb\Approved\Approved On: 19-Feb-2010 20:59

88. Lewis, D., et al., A Phase 2/3, Randomized, Double Blind, Comparative Trial of Azithromycin Plus Chloroquine Versus Mefloquine for the Treatment of Uncomplicated Plasmodium falciparum Malaria in Africa. , in 5th European Congress on Tropical Medicine and International Health. 2007: Amsterdam.

89. Chandra R, et al., A Phase 3, Randomized, Open-Label, Comparative Trial of Azithromycin plus Chloroquine versus Mefloquine for the Treatment of Uncomplicated Plasmodium falciparum Malaria in Africa, in 56th Annual Meeting of the American Society of Topical Medicine and Hygiene. 2007, Pfizer Inc: Philadelphia.

90. CDC, Sexually Transmitted Diseases Treatment Guidelines, 2006. MMWR, 2006. 55 (No. RR-11).

91. Most H, Clinical Trials of Antimalarial Drugs, in Internal Medicine in World War II, Coates JB, Editor. 1963, Office of the Surgeon General, Medical Department, United States Army: Washington DC.

92. Dorsey G, S.S., Clark TD, Njama-Meya D, Nzarubara B, Maiteki-Sebuguzi C, Dokomajilar C, Kamya MR, Rosenthal PH., Combination Therapy for Uncomplicated Falciparum Malaria in Ugandan Children, A Randomized Trial. JAMA 2007. 297(2): p. 2210-19.

93. Zongo I, D.G., Rouamba N, Dokomajilar C, Sere Y, Rosenthal PJ, Ouedraogo JB, Randomized Comparison of Amodiaquine plus Sulfadoxine Pyrimethamine, Artemether-Lumefantrine, and Dihydroartemisinin-Piperaquine for the Treatment of Uncomplicated Plasmodium falciparum Malaria in Burkina Faso. CID 2007. 45: p. 1453-61.

94. Sykes, A., et al., Azithromycin plus Artesunate versus Artemetherâ€□Lumefantrine for Treatment of Uncomplicated Malaria in Tanzanian Children: A Randomized, Controlled Trial. Clinical Infectious Diseases, 2009. 49(8): p. 1195-1201.

95. Hoepelmana I.M., Schneider M.M.E. Azithromycin: the first of the tissue-selective azalides. International Journal of Antimicrobial Agents. 1995(5): 145-167.

96. G. Foulds, R.A. Ferraina, H. G. Fouda, R. B. Johnson, A. M. Kamel, and R. M. Shepard, (1995) Azithromycin Concentrations in Gallbladder, hepatic tissue, and bile following a 5-day regimen in humans. Infectious disease and therapy (1995), 18:367-372.

090177e1811d3ccb\Approved\Approved On: 19-Feb-2010 20:59

Appendix 1. Rapid Diagnostic Testing for *P. falciparum*

NOW® ICT MALARIA

A rapid WHOLE BLOOD Immunochromatographic test (ICT) (U.S. Patent No(s): 5,877,028; 5,998,220; 6,017,767) for the qualitative detection of *Plasmodium falciparum* (P.f.), *Plasmodium vivax* (P.v.), *Plasmodium malariae* (P.m.) and *Plasmodium ovale* (P.o.) antigens.

For in vitro diagnostic use.

Test Principle

The NOW® ICT Malaria test is a rapid, in vitro immunodiagnostic test for the detection of circulating *Plasmodium falciparum* (P.f.) antigen and an antigen that is common to all four species of malaria, *Plasmodium falciparum* (P.f.), *Plasmodium vivax* (P.v.), *Plasmodium ovale* (P.o.) and *Plasmodium malariae* (P.m.) in whole blood. The test uses two antibodies that have been immobilized across the test strip. One antibody is specific for the histidine-rich protein II antigen of *P. falciparum* (P.f. HRPII). The other antibody is specific for an antigen that is common to P.f., P.v., P.m. and P.o.

A procedural control line is also immobilized across the test strip and will always appear in area C of the test window, if the test has been performed correctly. Whole blood (15µL) is applied to a sample pad impregnated with colloidal gold-labeled antibodies, which are directed against the malarial antigens. When a positive sample is applied, malarial antigens bind to the gold-conjugated antibodies in the sample pad. Reagent is then added which allows the immune complexes formed to migrate along the test strip where they are captured by the immobilized antibodies. When capture occurs, one or two pink lines will form in the test window. When a negative sample is applied only the control line will appear.

Acknowledgement

Binax, Inc. acknowledges F. Hoffman La Roche Corporation for the provision of antibodies used in this test.

Specimen Collection

Use an EDTA capillary tube, capable of delivering 15µl, or venous blood collected into EDTA tubes.

1. To obtain capillary blood via puncture of a finger, heel or other appropriate site, cleanse the area with a sterile swab and dry with a sterile pad. Use a lancet to puncture the skin and collect the blood directly into the capillary tube. Fill the entire capillary tube with blood and use immediately.

090177e1811d3ccb\Approved\Approved On: 19-Feb-2010 20:59

2. Collect venous blood, by the standard venipuncture procedure, into an EDTA tube. If the test cannot be performed immediately, the blood may be stored for up to three days at 2˚ to 8˚C.

Precautions and Warnings

- Optimal results will be obtained by strict adherence to this protocol. Reagents must be added carefully to maintain precision and accuracy. Treat used cards as biohazardous. Do not reopen or reuse test cards.
- Biological contamination of dispensing equipment, containers or reagents can lead to false results. Observe established precautions against microbiological and serological hazards in specimen handling, disposal and throughout all procedures.
- Do not use kits beyond their expiration date. Keep storage boxes dry.
- Store kits at 2˚ - 30˚C.
- Reagent A contains sodium azide as a preservative. Sodium azide is toxic and should, therefore, be handled carefully, avoiding ingestion or skin contact. It may react with lead or copper plumbing to form explosive metal azides. Flush with a liberal volume of water when disposing of unwanted reagent.
- Do not mix reagents from different kit lots.

Limitations of Procedure

The test is able to identify an infection caused by P.f. or one that is caused by either P.v., P.o. or P.m. The test cannot speciate a P.f. mixed infection, as the antibody used on test line 2 may be positive in patients with P.f., P.v., P.o., and P.m. Performance characteristics of this test have been evaluated using P.f. and P.v. samples only. Occasionally, residual P.f. HRPII antigen may be detected for several days following elimination of the parasite by anti-malarial treatment. Diagnosis should be made using the results of this test together with the other clinical and laboratory findings.

Kit Contents

- Individually packaged test cards;
- Capillary tubes;
- Malaria Reagent A;
- Product insert;
- Procedure card.

090177e1811d3ccb\Approved\Approved On: 19-Feb-2010 20:59

Materials Required but not Included

- Lancets;
- Sterile swabs.

Test Procedure

Ensure all test components are room temperature prior to use. Just prior to use, remove the test card from the pouch. Open the card and lay it flat on the work surface.

Using an EDTA capillary tube, collect blood by puncturing an accessible site (eg, finger or heel). Ensure that the capillary tube is completely filled. Alternatively, 15µl of blood may be collected using a tube of venous blood. Ensure blood sample is room temperature prior to use.

Slowly apply blood from the capillary tube to cover the entire PURPLE sample pad (See diagram). This is done by holding the capillary tube vertically and gently pressing the end against the purple pad in several places. The test will not require all of the blood that has been collected into the capillary tube. Once the purple pad is saturated, discard the capillary tube. If using a micro-pipette, apply 15µl of blood to the purple pad. Ensure that the blood sample is added slowly to the bottom of the purple sample pad. Do not add the blood sample all at once. Ensure that the sample is placed drop by drop at the bottom of the purple pad.

IMPORTANT: Incorrect addition of sample may lead to device failure.

Holding the Reagent A bottle vertically, apply two drops of Reagent A to the white pad immediately below where the blood was applied (See Diagram).

Apply four drops of Reagent A to the pad located at the top of the left hand side of the test card (See diagram).

Remove and discard the adhesive liner. Ensure that the adhesive on the right hand side of the test card is exposed.

Allow the blood sample to run up the full length of the test strip. Wait until the red lysed blood reaches the base of the white absorbent pad located at the top of the test strip (See Diagram).

Immediately close the card. To ensure good test flow, press very firmly along the entire area to the right of the window. Start timing.

Read the result through the viewing window at 10 minutes. High positive results may be visible sooner. Refer to the details given in this insert for test interpretation.

Positive Test Result infection: A positive test result is indicated by any visible line in the test window next to T1 together with a line in area C.

090177e1811d3ccb\Approved\Approved On: 19-Feb-2010 20:59

P.f. infection or a mixed infection (P.f., P.o., Pm., P.v.): A positive test result is indicated by any visible line in the test window next to T1 and T2, together with a line in area C.

P.v., P.m., P.o. or a mixed infection of all three: A positive test result is indicated by any visible line in the test window next to T2, together with a line in area C.

Negative Test Result

The test is negative if only the C line appears.

Invalid Test Result

The test is invalid if the C line does not appear. If this occurs, the test should be repeated using a new card.

090177e1811d3ccb\Approved\Approved On: 19-Feb-2010 20:59
